# Acid‐Responsive Nanobot‐Integrated Core‐Shell Microneedles Reprogram the Degenerative Annulus Fibrosus Microenvironment Through Epigenetic Suppression of Ferroptosis

**DOI:** 10.1002/advs.77829

**Published:** 2026-09-27

**Authors:** Lu Tan, Yan Zheng, Yue Lan, Zhuo Cheng, Siya Wang, Changqing Li, Yanqiu Wang, Minghan Liu

**Affiliations:** ^1^ Department of Orthopedics Xinqiao Hospital Army Medical University Chongqing China; ^2^ Chongqing Municipal Health Commission Key Laboratory of Precise Orthopedics Chongqing China; ^3^ Chongqing Yuyue Scientific Research Center for Pathology Jinfeng Laboratory Chongqing China; ^4^ School of Pharmacy and Bioengineering Chongqing University of Technology Chongqing China; ^5^ Department of Orthopedics Army 953 Hospital Army Medical University Shigatse Xizang Autonomous Region China

**Keywords:** acid‐responsive nanobot‐integrated core‐shell microneedle system, annulus fibrosus regeneration, ATF3 promoter methylation, DNMT3A‐ATF3‐SLC7A11 axis, ferroptosis resistance, microenvironment reprogramming

## Abstract

Annulus fibrosus (AF) rupture is a key structural event in intervertebral disc degeneration, but effective repair is hindered by the acidic, hypoxic and oxidative microenvironment of the avascular disc. Single‐cell transcriptomic reanalysis and clinical AF specimens identified ferroptosis‐associated redox imbalance as a prominent feature of advanced degeneration. Here, we developed an acid‐responsive nanobot‐integrated core‐shell microneedle system for staged annulus fibrosus repair. The shell layer released TA@MgO_2_ metal‐phenolic nanobots that consumed pathological H^+^, generated O_2_, and provided NIR‐amplified redox buffering, thereby normalizing the early degenerative microenvironment. The core layer enabled sustained quercetin release to reinforce anti‐ferroptotic and matrix‐preserving effects. Mechanistically, TMH/QG+NIR suppressed ferroptosis in AF cells by epigenetically silencing ATF3 through DNMT3A‐dependent promoter methylation, thereby relieving ATF3‐mediated repression of SLC7A11 and restoring GPX4‐dependent antioxidant defense. In a puncture‐induced degeneration model, this system alleviated ferroptotic injury, improved AF integrity, preserved disc height and enhanced biomechanical resilience. These findings identify the DNMT3A‐ATF3‐SLC7A11 axis as a therapeutically tractable ferroptosis‐regulatory pathway and establish a staged microenvironment‐reprogramming strategy for AF repair.

## Graphical abstract

An acid‐responsive core‐shell microneedle system normalizes the degenerative annulus fibrosus microenvironment by consuming pathological H^+^, generating O_2_, and providing NIR‐amplified redox buffering. Mechanistically, it epigenetically suppresses ferroptosis via DNMT3A‐dependent methylation of the ATF3 promoter, relieving ATF3‐mediated repression of SLC7A11 and restoring GPX4‐mediated antioxidant defense, thereby establishing a staged repair strategy for annulus fibrosus regeneration.

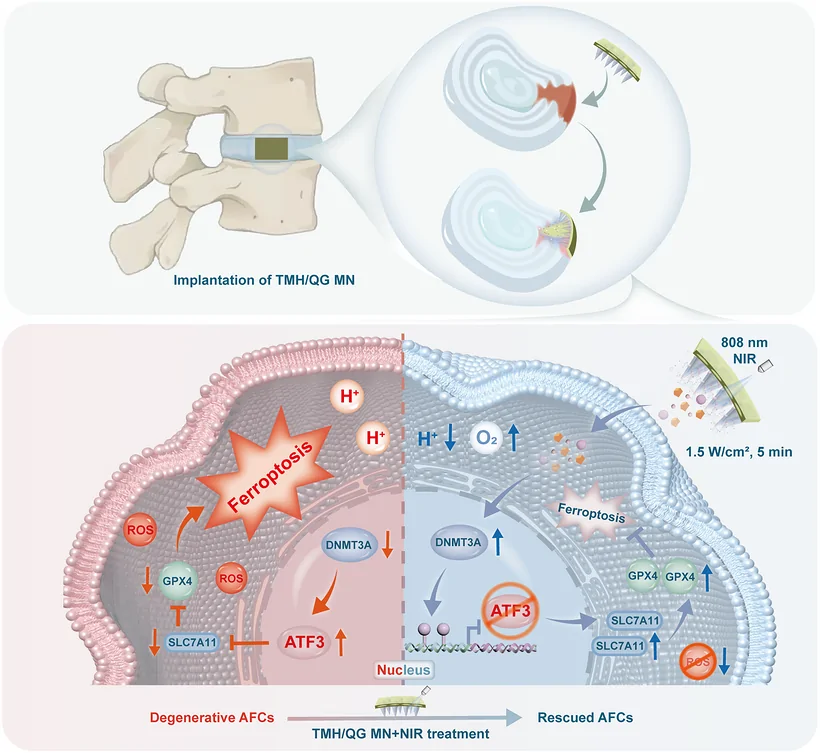

## 1 Introduction

Intervertebral disc degeneration (IDD) is a leading cause of low back pain (LBP) and affects more than 80% of adults worldwide. Current clinical interventions range from conservative anti‐inflammatory regimens to surgical fusion, yet none restore native tissue function because they either transiently relieve symptoms or permanently sacrifice spinal mobility [1, 2, 7, 8, 9, 10, 11]. At the center of IDD pathogenesis lies the progressive deterioration of the annulus fibrosus (AF), a highly organized collagen‐rich outer layer of the intervertebral disc (IVD) that maintains structural integrity and confines the nucleus pulposus (NP) [3, 4]. Damage of the AF triggers a cascade of biomechanical and biochemical disruptions, including collagen fragmentation and matrix metalloproteinase‐mediated extracellular matrix (ECM) depletion, events that collectively compromise load‐bearing capacity and accelerate disc collapse [4, 5, 6].

A critical barrier to AF repair arises from the unique microenvironmental pathophysiology of degenerated AF, which exhibits a hypoxic and acidic niche due to lactate accumulation from anaerobic glycolysis. This niche in turn triggers excessive reactive oxygen species (ROS) generation by stressed annulus fibrosus cells (AFCs), leading to degradation of collagen and proteoglycans [3, 12, 13]. Emerging evidence has identified ferroptosis as an iron‐dependent lipid peroxidation cascade that drives AFCs death and contributes to a self‐perpetuating cycle of ECM loss and mechanical failure. Although ferroptosis has emerged as an important cell‐death mechanism in disc degeneration, its cell‐subtype distribution in human degenerative AF and its association with clinical AF pathology remain insufficiently defined [14, 15, 16]. The above‐mentioned pathological events interact synergistically because acidosis exacerbates ROS production through mitochondrial dysfunction while ROS further lowers pH and intensifies ferroptosis. However, most existing therapeutic strategies target only a single pathological component and therefore fail to address the interconnected acidosis‐ROS‐ferroptosis cascade that evolves during AF degeneration [17, 18].

Conventional biomaterial platforms such as nanoparticles, hydrogels, and electrospun scaffolds have been explored for AF repair, but they generally lack sufficient tissue‐anchoring capability and cannot achieve sustained, localized delivery of therapeutic agents within the avascular AF lesion [19, 20, 21, 22, 23]. In contrast, microneedle (MN) systems offer distinct advantages for AF regeneration because they penetrate the dense AF tissue, provide mechanical interlocking, and deliver functional components directly to the injury site [24, 25, 26, 27]. Existing single‐layer microneedles (MNs) have improved local retention, mechanical anchorage or stimulus‐responsive release, but most remain passive delivery systems and do not exploit the acidic degenerative niche as an active trigger for staged microenvironmental normalization [3, 28, 29, 30, 31, 32, 33]. Moreover, most single‐layer MNs adopt mono‐therapeutic strategies that cannot address the multifactorial pathology of AF degeneration, and they also suffer from inadequate penetration depth, limited bioactivity preservation in the hostile degenerative niche, as well as short functional retention under dynamic physiological loading [34, 35]. An integrated core‐shell MN system is therefore required to match the mechanical properties of the AF, enable staged release of functional components, and maintain bioactivity throughout the repair process.

**FIGURE 1:**
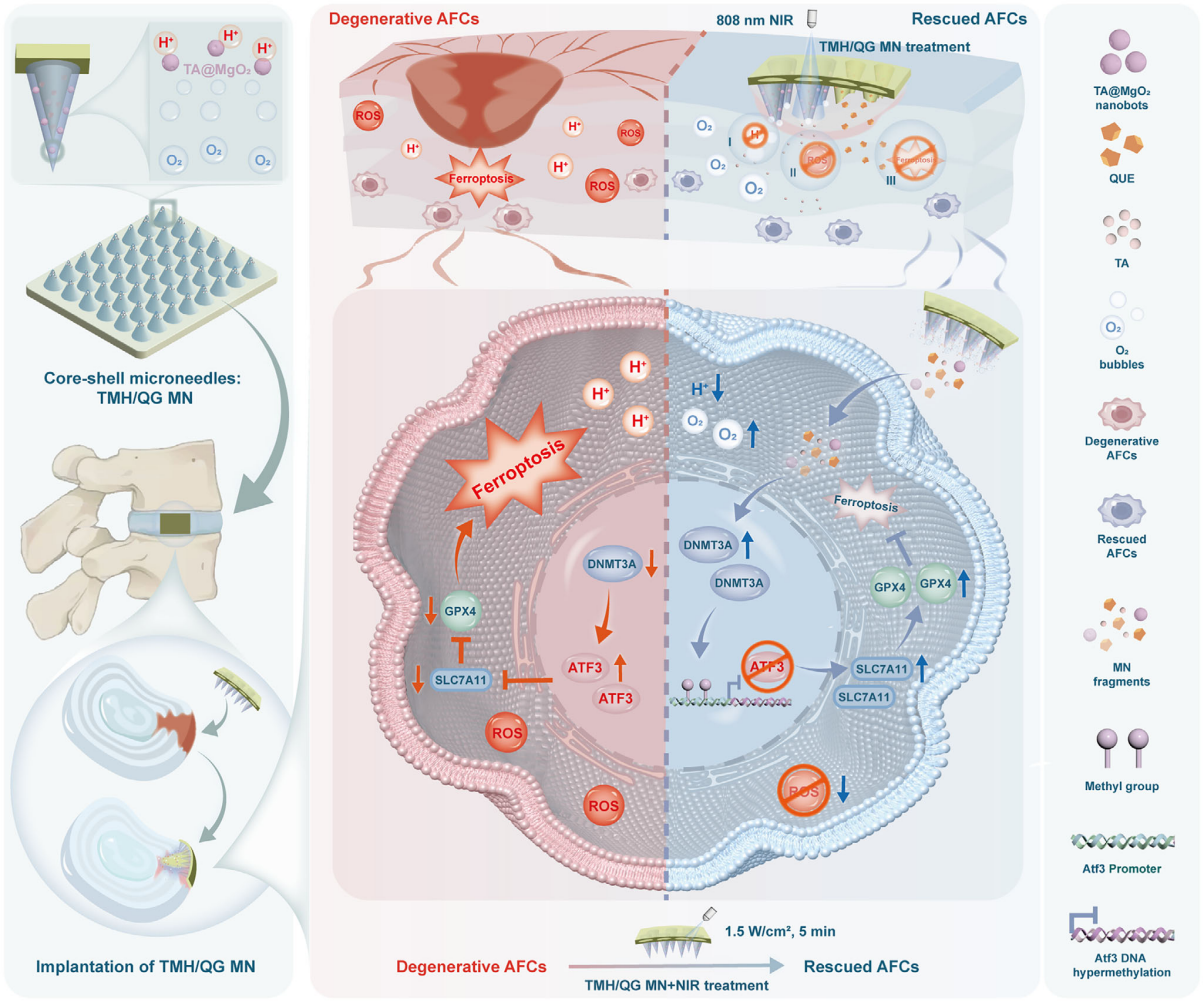
Schematic diagram of the nanobot‐integrated core‐shell microneedle system for sequential annulus fibrosus repair. In this work, we developed a nanobot‐integrated core‐shell microneedle system (TMH/QG MN) for annulus fibrosus repair, which presents a transformative strategy that couples self‐driven microenvironmental normalization with epigenetic modulation. Specifically, the shell layer contains metal‐phenolic nanobots that react with pathological H^+^ to generate O_2_, thereby alleviating hypoxia and acidosis while providing enzyme‐mimetic and photothermal‐enhanced antioxidant activity. The core layer sustains the release of quercetin, which exerts anti‐ferroptotic and anti‐inflammatory effects. Mechanistically, TMH/QG MN suppressed ferroptosis in annulus fibrosus cells by epigenetically silencing ATF3 through DNMT3A‐dependent promoter methylation, thereby relieving ATF3‐mediated repression of SLC7A11 and restoring GPX4‐dependent antioxidant defense. Collectively, these actions alleviated ferroptotic injury, restored matrix homeostasis, enhanced biomechanical resilience, and ultimately achieved annulus fibrosus repair.

To address these requirements, we designed an acid‐responsive nanobot‐integrated core‐shell microneedle system (TMH/QG) that uses pathological H^+^ accumulation as an endogenous trigger to initiate early microenvironmental normalization to enable sequential and synergistic AF regeneration through structured material and biological programming (Figure 1). The shell layer (TMH) consists of a mechanically enhanced hyaluronic acid‐based matrix (HA‐ADH) loaded with an acidic microenvironment‐driven self‐propelling nanobot composited of tannic acid (TA)@magnesium peroxide (MgO_2_). This TA@MgO_2_ nanobot exploits the acidic AF milieu to generate asymmetric propulsion because MgO_2_ reacts with H^+^ to produce O_2_, which simultaneously alleviates hypoxia and raises local pH. Specifically, the tannic acid (TA) component of the metal‐phenolic network exhibits multi‐enzyme mimetic activities that disrupt ROS cascades at multiple nodes. Under near‐infrared (NIR) irradiation, TA@MgO_2_ further enables programmable photothermal amplification that provides on‐demand and pulsatile antioxidant boosting, thereby significantly accelerating ROS decomposition and offering mild photothermal therapy to promote AF repair in a precisely controllable manner [36]. Following this initial microenvironmental remediation, the inner core layer (QG) is composed of quercetin (QUE)‐encapsulated β‐cyclodextrin‐modified gelatin methacryloyl (GelMA‐β‐CD) via host‐guest interactions, which sustains the release of QUE to directly suppress ferroptosis and promote ECM reconstruction. Moreover, human AF single‐cell transcriptomic reanalysis and clinical specimen validation identified ferroptosis‐associated redox imbalance as a prominent feature of severe AF degeneration. Based on this disease context, TMH/QG was designed to target the acidic and oxidative degenerative niche, thereby mitigating the acidosis‐ROS‐ferroptosis‐associated cascade and restoring AF structural and biomechanical integrity. This core‐shell microneedle system integrates self‐driven microenvironmental normalization, programmable photothermal amplification and DNA methylation‐mediated epigenetic suppression, distinguishing it from single‐target therapies and aligning therapeutic actions with the dynamic progression of AF degeneration, which shifts the AF treatment from single‐target intervention to phased and programmable tissue regeneration, establishing a new therapeutic framework for IDD management.

## 2 Results

### 2.1 Human Degenerative AF Exhibits Ferroptosis‐Associated Redox Imbalance

**FIGURE 2:**
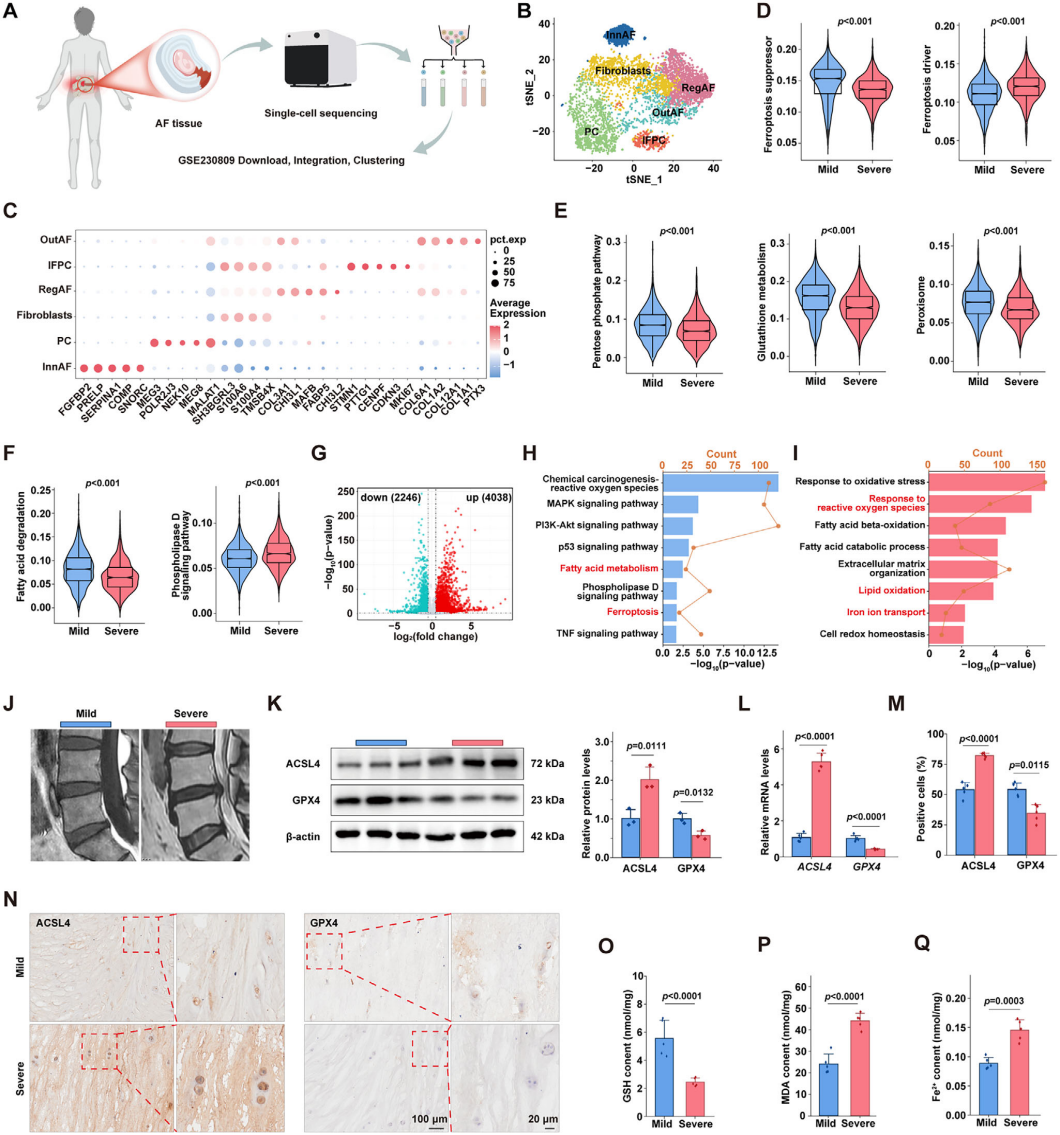
Degenerative AF is characterized by redox imbalance and ferroptosis activation. (A) Schematic illustration of single‐cell RNA‐seq in human degenerative annulus fibrosus tissues. (B) The tSNE plot visualizes six distinct clusters of human AF cells from intervertebral disc samples. (C) Dot plot displaying the marker genes specific to each AF cell sub‐cluster (mild = Pfirrmann II‐III, severe = Pfirrmann IV‐V). (D) The scores of ferroptosis suppressors and drivers were calculated using the AddModuleScore function. (E) Scores for the pentose phosphate pathway, glutathione metabolism, and peroxisome function were computed to assess the metabolic and antioxidant status of AF cells. (F) Fatty acid degradation and phospholipase D signaling pathway activities were evaluated to examine lipid metabolism and membrane remodeling in AF cells. (G) The volcano plot depicts 6284 DEGs between mild (*p* < 0.001) and severe (*p* < 0.001) AF samples. (H,I) Enrichment analysis of DEGs was performed to identify significantly enriched biological pathways in mild and severe AF samples. (J) MRI scans of human intervertebral discs from mild and severe degeneration groups. (K,L) Relative protein and mRNA expression levels of the key ferroptosis regulators ACSL4 and GPX4 in human mild and severe degenerative disc tissues. The corresponding uncropped blot images are provided in Table S1. (M‐N) IHC staining and quantification of positive cells for ACSL4 and GPX4 in mild and severe degenerative IVD samples. Scale bar: 20 µm. (O–Q) GSH content, MDA content, and Fe^2+^ levels in mild and severe degenerative IVD samples. Data are presented as mean ± SD (n ≥ 3), and *p*‐values were calculated using two‐tailed Student's *t*‐test [(D), (E), (F), (K), (L), (M), (O), (P), and (Q)].

The AF is essential for the normal biomechanical function of the IVD but exhibits a limited repair capacity once ruptured during IDD [37]. To investigate pathogenic factors contributing to IDD, we performed single‐cell RNA sequencing (scRNA‐seq) public dataset reanalysis on human AF tissues representing mild (Grade II and III) and severe (Grade IV and V) degenerative stages (GSE230809) (Figure 2A). Unsupervised clustering identified six distinct cell populations at a resolution of 0.4, which were visualized using t‐distributed stochastic neighbor embedding (*t*‐SNE) (Figure 2B). Systematic annotation based on differentially expressed marker genes identified these clusters as OutAF, inner fibrous progenitor cells (IFPC), RegAF, Fibroblasts, progenitor cells (PC) and InnAF (Figure 2C). Using this cellular map, we quantified ferroptosis‐related activity across AFC subpopulations by calculating module scores for curated ferroptosis suppressor and driver gene sets in mild and severe degeneration samples. As shown in Figure 2D, the severe degeneration group exhibited significantly lower ferroptosis‐suppressor scores than the mild group, whereas ferroptosis driver scores were elevated across all AFCs clusters. Having identified a marked imbalance in ferroptosis suppressor and driver scores in mild and severe degenerative groups, we next investigated the mechanistic basis for this dysregulation. Pathway scoring further indicated that the pentose phosphate pathway, glutathione metabolism and peroxisome‐related programs were reduced in severe degeneration, whereas phospholipase D signaling was increased compared with mild degeneration (Figure 2E,F). Based on these alterations at the transcriptional level, we compared the transcriptional data from the mild sample to that of the severe sample to identify 6284 differentially expressed genes (DEGs), which contained 2246 downregulated genes and 4038 upregulated genes (Figure 2G). Functional enrichment analysis showed that these DEGs were enriched in biological processes related to fatty acid metabolism, ferroptosis, response to reactive oxygen species (ROS), lipid oxidation and iron ion transport (Figure 2H,I).

To experimentally validate the bioinformatic predictions, we analyzed human AF tissues from patients with mild and severe IDD (Figure 2J) using reverse transcription quantitative PCR (RT‐qPCR) and western blot. The transcript level of the ferroptosis driver Acyl‐CoA synthetase long‐chain family member 4 (*ACSL4*) was elevated 4.67‐fold in severe degeneration samples, whereas the expression of the ferroptosis suppressor glutathione peroxidase 4 (*GPX4*) was reduced by 59.10% (Figure 2L). Consistent with these transcriptional changes, protein levels of ACSL4 and GPX4 showed corresponding alterations between degeneration groups (Figure 2K). We next evaluated ferroptosis‐associated marker expression by immunohistochemical (IHC) staining of AF tissues from different degeneration groups (Figure 2N). Quantitative analysis revealed that severe degeneration AF contained 1.52‐fold more ACSL4‐positive cells while showing a 36.19% reduction in GPX4‐positive cells compared to the mild degeneration group (Figure 2M). Having established this molecular and histopathological profile of ferroptosis, we next investigated the consequent functional redox imbalance and iron dysregulation by measuring the concentrations of malondialdehyde (MDA), glutathione (GSH) and ferrous ions (Fe^2+^) of AF from different groups, which are direct indicators of lipid peroxidation, antioxidant capacity and iron accumulation, respectively. Compared to the mild degeneration group, severe degeneration AF exhibited a reduction in GSH concentration from 5.55 to 2.44 nmol/mg, alongside an increase in MDA content from 23.98 to 44.01 nmol/mg (Figure 2O,P). Furthermore, Fe^2+^ concentration was approximately 1.64‐fold higher in the severe group than in the mild group (Figure 2Q). These biochemical findings consistently supported the preceding molecular and histopathological data, confirming that redox imbalance and ferroptosis activation represent hallmark pathological features during human IDD progression.

### 2.2 TA@MgO_2_ Nanobots Enable Acid‐Responsive Oxygen Generation and NIR‐Amplified Redox Buffering

**FIGURE 3:**
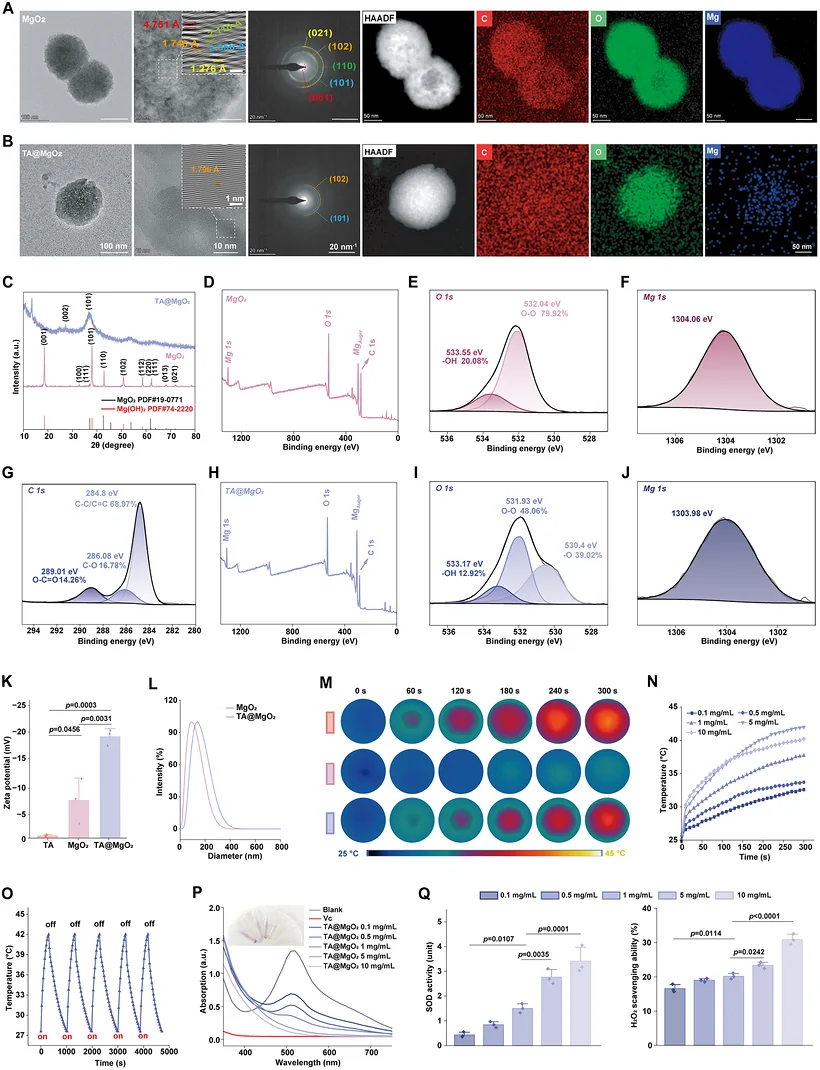
Characterization of acid‐activated oxygen‐generating nanobots with self‐propulsive potential. (A,B) TEM images, HRTEM images, SAED patterns, and corresponding EDS elemental mappings of MgO_2_ and TA@MgO_2_. Scale bar: 50 nm. (C) XRD patterns of MgO_2_ and TA@MgO_2_. (D–J) XPS spectra survey characterizing the chemical composition and elemental oxidation states of MgO_2_ and TA@MgO_2_, with high‐resolution XPS spectra of characteristic elements: O 1s and Mg 1s for MgO_2_ and O 1s, Mg 1s, and C 1s for TA@MgO_2_. (K) Zeta potential values of TA, MgO_2_ and TA@MgO_2_. (L) DLS measurements of MgO_2_ and TA@MgO_2_. (M) Infrared thermal images of TA, MgO_2_ and TA@MgO_2_ under NIR irradiation. (N) Temperature curves of TA@MgO_2_ at different concentrations under NIR irradiation (1.5 W/cm^2^). (O) Photothermal stability of TA@MgO_2_ evaluated via cyclic NIR irradiation tests (1.5 W/cm^2^, 5 min on/off). (P) UV–vis absorption spectra and digital photographs of DPPH free radicals treated with TA@MgO_2_ at different concentrations. (Q) Quantitative evaluation of the multi‐enzyme mimetic activities of TA@MgO_2_: SOD‐like activity and H_2_O_2_ scavenging capacity at different concentrations. Data are presented as mean ± SD (n ≥ 3), and *p*‐values were calculated using one‐way ANOVA with Tukey's multiple comparisons test [(K) and (Q)].

TA contains multiple phenolic hydroxyl‐substituted benzene rings and exhibited a characteristic concentration‐dependent ultraviolet (UV) absorption peak at ∼290–295 nm (Figure S1A). UV–vis spectroscopy confirmed the presence of this signature absorption only in samples of free TA and the TA@MgO_2_ (Figure S1B). The TA‐to‐MgO_2_ mass ratio was optimized through a gradient exploration (Figure S1C), and the 1:1 ratio was selected for subsequent experiments as it yielded the highest drug loading content of 17.16% (Figure S1D). To further detect the morphology of nanoparticles, we employed transmission electron microscopy (TEM) to observe the TA reacting with MgO_2_ to form TA@MgO_2_ metal‐phenolic network (MPN) with self‐propelled potential. Figure 3A,B revealed representative TEM images of MgO_2_ and TA@MgO_2_, respectively, which indicated that the pure MgO_2_ exhibited the typical spherical shape, and the corresponding energy‐dispersive spectroscopy (EDS) mapping also revealed similar morphology and homogeneous distribution of carbon (C), oxygen (O) and magnesium (Mg) within the MgO_2_ and TA@MgO_2_ structure. Besides, the high‐resolution transmission electron microscope (HRTEM) and selected‐area electron diffraction (SAED) images of MgO_2_ and TA@MgO_2_ showed the well‐crystallized MgO_2_ with typical lattice distances that are assigned to the typical planes of MgO_2_. It suggested that the modification of TA did not induce the phase transformation and lattice distortion in the MgO_2_ core, which preserves the crystallographic integrity of MgO_2_ to ensure the retention of its self‐propulsion capability in the acidic microenvironment. The above‐mentioned results were also supported by the result of X‐ray diffraction (XRD) in Figure 3C. Subsequently, we employed X‐ray photoelectron spectroscopy (XPS) to verify the chemical composition of MgO_2_ and TA@MgO_2_, demonstrating that the successful surface modification of TA in MgO_2_, rather than a physical mixture, and TA@MgO_2_ retained the MgO_2_ core structure (Figure 3D–J). Moreover, the zeta potential value of MgO_2_ decreased from −6.9 mV to −18.68 mV for TA@MgO_2_ (Figure 3K), while the diameter increased from 94.35 nm to 144.73 nm (Figure 3L), not only verifying the successful surface engineering but also endowing the TA@MgO_2_ nanobots with enhanced electrostatic stabilization.

Based on the NIR treatment induced the photothermal conversion property of MPN, we assessed its photothermal conversion performance with different concentrations. As shown in Figure 3N and Figure S1F, TA@MgO_2_ could address a mild thermal treatment with a typical concentration‐ and NIR laser power‐dependent performance. Besides, the temperature reached approximately 42°C each NIR laser irradiation cycle and returned to baseline upon laser removal (Figure 3O), validating that TA@MgO_2_ can reproducibly provide consistently mild thermal stimulation for IDD therapy. We compared the photothermal efficacy of TA@MgO_2_ and its individual components (MgO_2_ and TA) using infrared thermography. Since the MPN structure of TA@MgO_2_ exhibited markedly superior photothermal conversion compared to pure MgO_2_ and TA (Figure 3M), the corresponding quantitative analysis also demonstrated that the final temperature of TA@MgO_2_ increased to 42.7°C, which was 1.43‐fold higher than that of pure MgO_2_ (p = 0.0002, Figure S1E). The above results suggested that the TA@MgO_2_ nanobots exhibit pronounced photothermal properties, which were ascribed to the coordination between TA and Mg^2+^ that created a charge‐transfer complex that enhanced NIR absorption and facilitated highly efficient non‐radiative relaxation, thereby generating the thermal output essential for mild photothermal therapy, resulting in TA@MgO_2_ functioning as a potent photothermal transducer upon NIR irradiation during the IDD therapy.

Because degenerative AF is characterized by elevated ROS levels, we employed the DPPH assay to quantitatively assess the free radical scavenging capability of TA@MgO_2_. Visual assessment revealed a pronounced concentration‐dependent scavenging effect, as evidenced by the gradual color change of the reaction mixture from purple to yellow as the concentration of TA@MgO_2_ increased from 0.1 to 10 mg/mL, and the corresponding ultraviolet absorption peak at 517 nm gradually decreased (Figure 3P). The quantitative statistical results showed that the DPPH scavenging ability of 5 mg/mL and 10 mg/mL TA@MgO_2_ was 1.81‐times (*p* < 0.0001) and 1.91‐times (*p* < 0.0001) higher than that of 0.1 mg/mL sample, respectively (Figure S1G). Furthermore, TA@MgO_2_ exhibited significant superoxide dismutase (SOD)‐mimetic activity in a concentration‐dependent manner. Specifically, the calculated SOD‐like activity of 0.1 mg/mL, 0.5 mg/mL, 1 mg/mL, 5 mg/mL and 10 mg/mL TA@MgO_2_ were 0.44, 0.84, 1.51, 2.77, and 3.42 units, respectively (Figure 3Q). Besides, TA@MgO_2_ also demonstrated a potent ability to decompose hydrogen peroxide (H_2_O_2_). After 24 h of co‐incubation, the H_2_O_2_ scavenging rate increased from 16.6% to 30.9% as the TA@MgO_2_ concentration was raised from 0.1 to 10 mg/mL (Figure 3Q). These results demonstrated that TA@MgO_2_ nanobots exhibited stable acid‐responsive oxygen‐generating and redox‐buffering properties. Under NIR irradiation, the TA‐Mg^2+^ metal‐phenolic coordination structure enabled reproducible mild photothermal conversion around the therapeutic hyperthermia range, while maintaining concentration‐dependent radical scavenging, SOD‐like activity and H_2_O_2_ decomposition capacity.

### 2.3 Core‐Shell TMH/QG Microneedles Achieve Staged Release, AF Penetration and Microenvironment‐Responsive Degradation

Figure S2 revealed that synthesis processes of the dihydrocaffeic acid (HCA) and hydrazide (ADH)‐modified hyaluronic acid (HA) as the shell microneedle precursor (HA‐ADH‐HCA), and the carboxymethyl‐β‐cyclodextrin (CM‐β‐CD) modified GelMA loading QUE as the core microneedle precursor (GelMA‐β‐CD@QUE), respectively. As shown in Figure S3A, the Fourier transform infrared spectrum (FTIR) exhibited absorption peaks at 1234, 1312, 1560, and 1630 cm^−1^, which were ascribed to the C‐O stretch of phenolic group, C‐N stretch of hydrazone bond, C = C aromatic stretch of HCA, and C = N stretch of hydrazone bond, respectively, demonstrating the successful synthesis of HA‐ADH‐HCA. Meanwhile, ^1^H nuclear magnetic resonance (^1^H NMR) spectroscopy in Figure S4A–C confirmed the successful synthesis of the HA‐ADH, as evidenced by the appearance of a new methylene peak at 2.3 ppm corresponding to ADH. Characteristic proton signals verified subsequent conjugation with HCA to form HA‐ADH‐HCA at 2.46 ppm (ADH methylene), 2.61 ppm (HCA α‐methylene), 2.81 ppm (HCA β‐methylene), and 6.75 ppm (HCA aromatic protons) (Figure S4D).

The FTIR spectrum of GelMA exhibited characteristic absorption bands at 3292 (N‐H/O‐H stretch), 1629 (amide I and C = C stretch), and 1533 cm^−1^ (amide II). A noticeable intensity increased at 1629 cm^−1^ compared to pure gelatin provided initial evidence of the synthesis of GelMA (Figure S3B). Further confirmation was obtained from ^1^H NMR analysis. While the spectrum of gelatin showed proton signals at 1.2–1.8 ppm (methylene groups) and 4.35 ppm (proline α‐H) in Figure S4E, the GelMA spectrum displayed new resonances at 1.84 ppm (MA methyl group), 5.57 ppm (MA vinyl proton), 3.05 ppm (glycine α‐methylene), and 3.36 ppm (lysine ε‐methylene) in Figure S4F, unequivocally verifying the successful modification. The successful conjugation of CM‐β‐CD to GelMA via amide bond formation was confirmed by FTIR and ^1^H NMR spectroscopy. The FTIR spectrum of CM‐β‐CD exhibited a characteristic peak at 1614 cm^−1^, corresponding to the asymmetric stretching vibration of carboxylate anions (Figure S3C). Following conjugation, the GelMA‐β‐CD spectrum showed the essential absorption peaks at 1550  (amide II band) and 1024 cm^−1^ (C‐O‐C/C‐O stretching vibrations from β‐CD). A noticeable blue shift of the amide II band from 1533 cm^−1^ in pure GelMA to 1550 cm^−1^ in the conjugate suggested the formation of new amide linkages (Figure S3B,D). Further evidence was provided by ^1^H NMR (Figure S4G–H), in which the GelMA‐β‐CD spectrum displayed proton resonances at 1.82 ppm (MA methyl groups), 3.63 ppm (cavity protons of β‐CD), 5.14 ppm (anomeric protons of β‐CD), and 5.28 ppm (MA vinyl protons), which all proved the successful synthesis of GelMA‐β‐CD. Since the extended conjugated scaffold and the catechol moiety of QUE, which exhibited the characteristic UV absorption at 375 nm and performed a concentration‐dependent property (Figure S5A). Therefore, the UV–vis spectroscopy in Figure S5B confirmed that only QUE and the GelMA‐β‐CD@QUE displayed this absorption peak at 375 nm. Based on the QUE calibration curve in Figure S5C, the mass feed ratio of 1:1 (GelMA‐β‐CD to QUE) was determined to achieve a drug loading content of 9.46%, which was employed in further experiments (Figure S5D).

**FIGURE 4:**
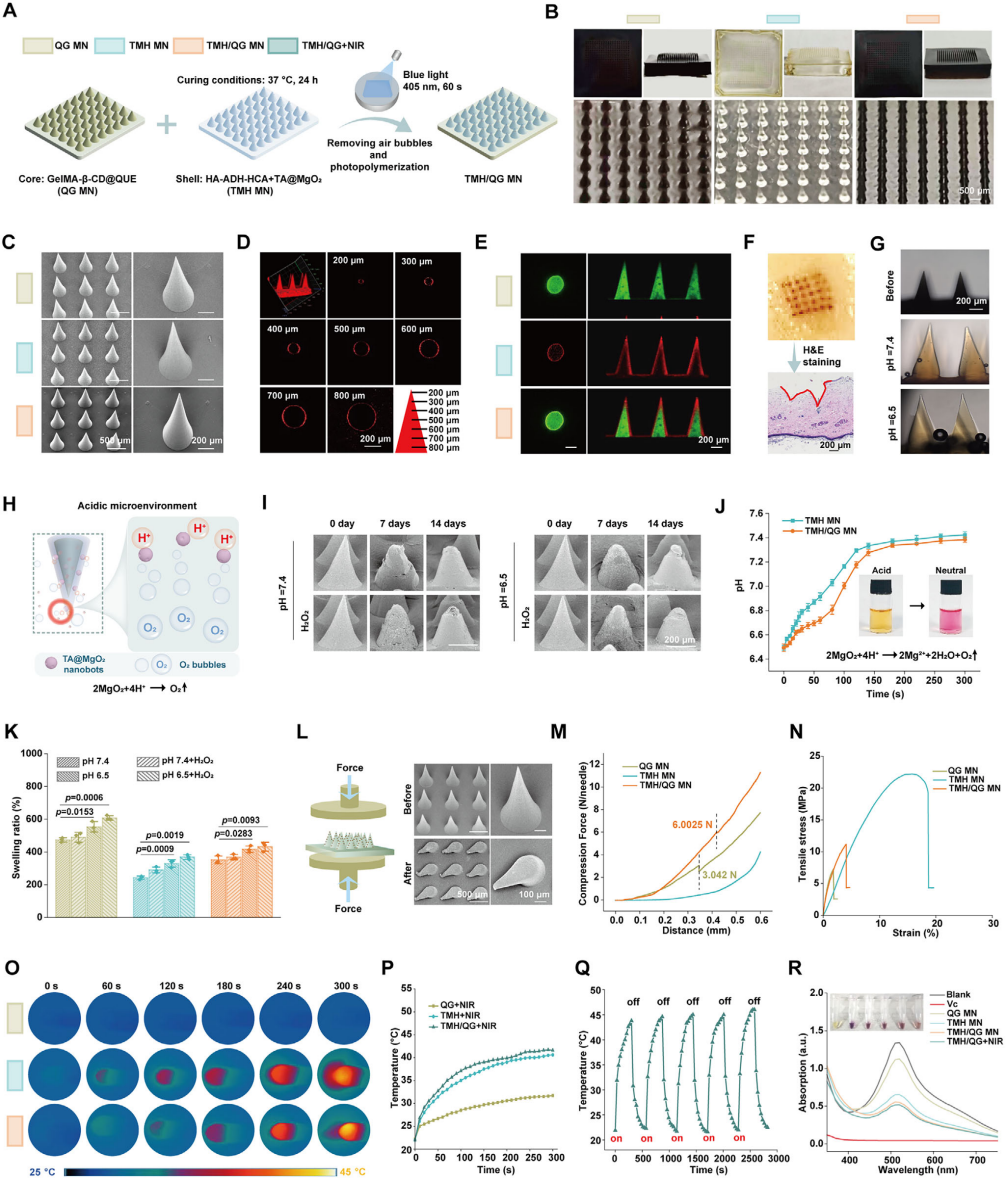
Characterization of core‐shell TMH/QG microneedles. (A) Schematic illustration of the synthesis of the core‐shell TMH/QG MN system. (B) Digital photographs of QG MN, TMH MN, and core‐shell TMH/QG MN, demonstrating the uniform surface array morphology and macroscopic structure of MNs. (C,D) Morphological characterization of the MNs. Scale bar: 200 µm. (E) CLSM image of the core‐shell structure, with the core labeled with FITC (green) and the shell labeled with Cy5 (red). Scale bar: 200 µm. (F) Digital photographs and H&E‐stained histological sections demonstrating successful skin penetration by the TMH/QG MN array. Scale bar: 200 µm. (G,H) Schematic and corresponding morphological changes in TMH/QG MN before and after incubation in PBS at pH 6.5 and 7.4, respectively. Scale bar: 200 µm. (I) SEM images visualizing the time‐dependent degradation behavior of TMH/QG MN in four simulated microenvironments (pH = 7.4, pH=6.5, pH=7.4+H_2_O_2_ and pH=6.5+H_2_O_2_). Scale bar: 200 µm. (J) pH‐responsive color change of TMH and TMH/QG MNs in an acidic medium containing phenolphthalein. (K) Swelling ratios of QG, TMH, and TMH/QG MN in four simulated microenvironments. (L) Schematic and corresponding SEM images of TMH/QG MN before and after mechanical compression. Scale bar: 100 µm. (M,N) Compression stress‐strain curves and tensile stress‐strain curves of the QG, TMH and TMH/QG MNs. (O) Infrared thermal images of QG, TMH and TMH/QG MNs under NIR irradiation. (P) Temperature curves of QG, TMH and TMH/QG MNs under NIR irradiation. (Q) Photothermal stability of the TMH/QG MN evaluated via cyclic NIR irradiation tests (1.5 W/cm^2^, 5 min on/off). (R) UV–vis absorption spectra and digital photographs of DPPH free radicals treated with QG, TMH, TMH/QG and TMH/QG+NIR MN. Data are presented as mean ± SD (n ≥ 3), and *p*‐values were calculated using one‐way ANOVA with Tukey's multiple comparisons test (K).

Although single‐layer MNs have improved local delivery for intervertebral disc repair, they are generally limited in temporally separating early microenvironmental correction from delayed therapeutic reinforcement. To address these challenges, we employed the HA‐ADH‐HCA to integrate the TA@MgO_2_ nanobots with NIR‐induced the pulsatile antioxidant boosting, and GelMA‐β‐CD@QUE was used to form the core‐layer MN (QG MN) by lithium phenyl‐2,4,6‐trimethylbenzoylphosphinate (LAP) to develop the core‐shell microneedle system (TMH/QG MN) (Figure 4A). Due to the characteristic of sequential degradation, the shell‐layer TMH MN can generate asymmetric thrust to drive TA@MgO_2_ nanobots' movement in the acidic microenvironment of the degenerated AF to improve hypoxia and reducing ROS levels, and the core‐layer QG MN successively released QUE to restore cellular viability to achieve the purpose of AF repair. The macroscopic images of the shell‐layer, core‐layer, and shell‐core MN array all revealed well‐defined structural integrity and precise geometrical morphology (Figure 4B). Higher‐resolution analysis via scanning electron microscopy (SEM) and confocal laser scanning microscopy (CLSM) indicated that the needles had a pyramidal contour, smooth surface topography with intact and sharply pointed tips, with a height of 800 µm and a base diameter of 350 µm (Figure 4C,D). Meanwhile, the CLSM images revealed that the shell layer of the TMH/QG MN showed red fluorescence, while the core exhibited green fluorescence, indicating that the core‐shell structure was successfully fabricated (Figure 4E). Besides, the shell‐core TMH/QG MN array exhibited a large aspect ratio to ensure that its sharp needle tips could easily penetrate and fix in the tissue (Figure 4F and Figure S7D), revealing the potential to achieve precise drug delivery in the AF area.

Upon exposure to an acidic medium (pH 6.5), the shell‐layer MN preferentially degraded and released TA@MgO_2_, which generated O_2_ and consumed H^+^, increasing the pH of the model solution from 6.5 to 7.4 and restoring the red color of phenolphthalein‐containing medium (Figure 4G–J). These findings indicate the capacity of TMH MNs to buffer acidic and hypoxic cues in a degenerative AF‐like microenvironment. To further assess the degradation performance, we placed the shell‐core TMH/QG MN into normal (pH 7.4), acidic (pH 6.5), ROS‐enriched (pH 7.4+H_2_O_2_) and mimic degenerative AF (pH 6.5+H_2_O_2_) microenvironments, respectively, and recorded their morphology alterations at different periods. As shown in Figure 4I, SEM imaging revealed the time‐dependent degradation of the core‐shell TMH/QG MN across the four above‐mentioned distinct microenvironments, characterized by progressive blunting of the needle tips and a gradual reduction in height. In the degenerative AF‐mimicking medium (pH 6.5 + H_2_O_2_), the height of core‐shell TMH/QG MNs decreased from 674 µm to 557 µm after 7 days and to 425.67 µm after 14 days, corresponding to degradation rates of 47.52% and 62.63%, respectively (Figure S6A,B). In addition, the degradation performances of TMH/QG MN under four simulated cell‐culture microenvironments (Figure S6C) were consistent with those observed in the above‐mentioned PBS‐based buffer systems in Figure S6A–B. Under this condition, the released amounts of TA and QUE reached 123.79 and 35.67 µg, respectively (Figure S6D,E), supporting microenvironment‐responsive degradation and staged drug release. To comprehensively assess swelling behavior, we also placed the shell‐layer TMH MN, core‐layer QG MN and shell‐core TMH/QG MN samples in four distinct microenvironments. As shown in Figure 4K, the swelling ratios of TMH/QG MN in the four above‐mentioned distinct microenvironments were 351.50%, 368.21%, 416.92% and 431.96%, respectively. The core‐shell architecture was designed to separate early microenvironmental neutralization from delayed anti‐ferroptotic drug release. In an acidic and ROS‐enriched medium mimicking degenerative AF, TMH/QG MNs showed accelerated shell degradation, H^+^ consumption, sustained TA and QUE release, and swelling‐mediated defect filling, supporting a staged therapeutic sequence rather than passive co‐delivery.

To ensure effective penetration of the AF for therapeutic delivery, we quantitatively measured the penetration force of individual needles from the shell‐layer TMH MN, core‐layer QG MN and shell‐core TMH/QG MN through the miniature multi‐scale in situ mechanical test system IBTC‐300 (Figure 4L). The results in Figure 4M indicated that the penetration force of TMH/QG MN reached 6.0025 N/needle, substantially exceeding the typical force required for tissue penetration (∼0.1–3 N), which proved that TMH/QG MN had the potential to successfully penetrate the AF tissue and maintain its integrity for subsequent treatment. In addition, the tensile properties of microneedles were also critical for AF therapy. The tensile curves (Figure 4N) and quantitative analysis (Figure S7A–C) revealed that shell layer and core layer contributed high strength and toughness for shell‐core TMH/QG MN, respectively, and their tensile modulus, fracture strength, and the elongation at break were 172.40 MPa, 571.58 MPa and 5.08%, respectively.

The above results proved that TA@MgO_2_ exhibited the concentration‐ and power‐dependent photothermal conversion performance, thus we further assessed the photothermal conversion ratio and stability of the shell‐layer TMH MN, core‐layer QG MN and shell‐core TMH/QG MN. As shown in Figure 4P, the shell‐layer TMH MN and shell‐core TMH/QG MN contained TA@MgO_2_, so that the relevant temperatures increased from 22°C to 40.6°C and 41.6°C under NIR laser irradiation (1.5 W/cm^2^, 5 min), respectively. Meanwhile, the shell‐core TMH/QG MN also revealed good photothermal stability, in which the temperature was maintained at ∼44.6°C–46°C during five NIR on‐off cyclic treatments (Figure 4Q). The visualized results from the infrared thermography in Figure 4O and Figure S5F both showed a similar trend. Compared to the core‐layer QG MN (30.0°C) without any photothermal conversion agents, the temperatures of the shell‐layer TMH MN (41.1°C) and shell‐core TMH/QG MN (40.9°C) containing TA@MgO_2_ were almost 1.38‐fold (*p* < 0.001) and 1.36‐fold (*p* < 0.001) higher than that of the QG MN group, respectively. These results collectively verified that shell‐core TMH/QG MN performed the necessary photothermal properties to provide the mild photothermal therapy for degenerative AF repair.

In addition, we also measured all aspects of the antioxidant abilities of the microneedles. The color alteration of Figure 4R showed that all MN samples had effective DPPH elimination capacities, with the relevant UV–vis spectra exhibiting a significant decrease in absorption intensity at 517 nm. The corresponding quantitative analysis illustrated that the TA@MgO_2_ and its induced the photothermal effect of the shell‐core TMH/QG MN could efficiently eliminate ROS (Figure S6F), which was also supported by the results of SOD‐like activity (Figure S6F) and H_2_O_2_ clearance capacity (Figure S6F) measurements, suggesting that shell‐core TMH/QG MN with NIR treatment had the promising potential to mitigate oxidative stress and delay AF degeneration.

### 2.4 TMH/QG+NIR Attenuates Ferroptosis and Restores Matrix Homeostasis in Oxidatively Stressed AFCs

**FIGURE 5:**
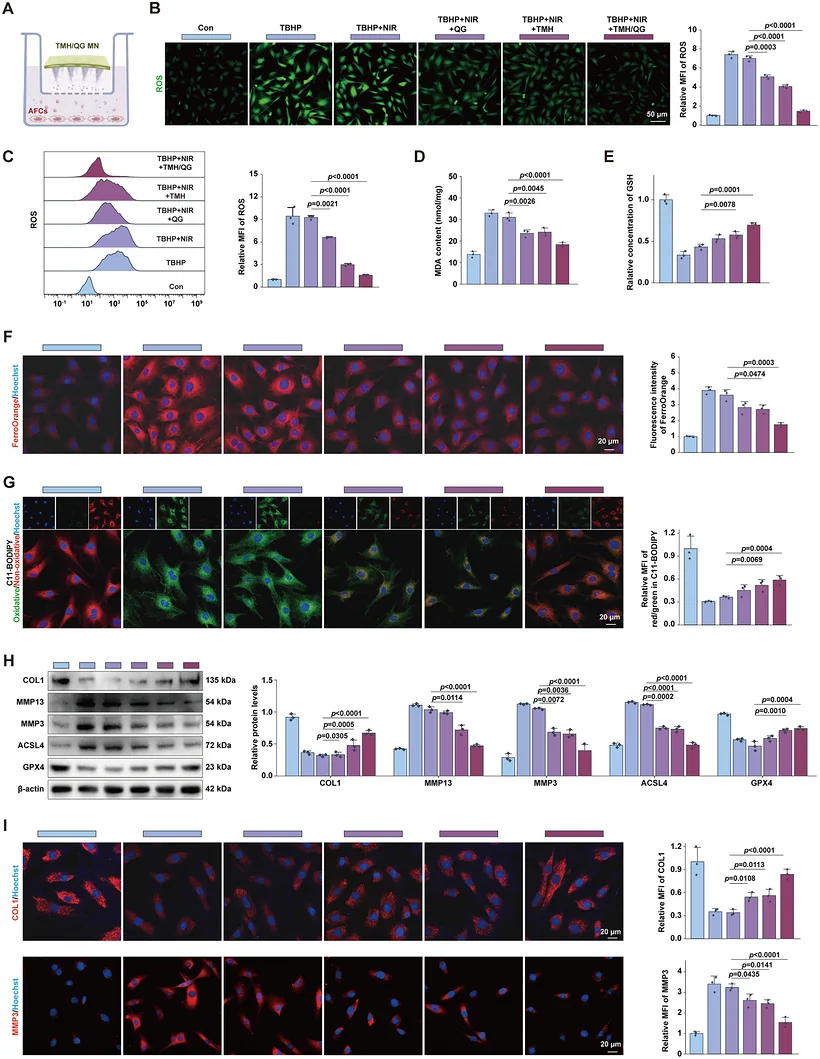
TMH/QG+NIR attenuates ferroptosis and matrix degradation in TBHP‐treated AFCs. (A) Schematic illustration of AF cells co‐culture system. (B) DCFH‐DA fluorescence staining images and quantitative analysis of TBHP‐pretreated AFCs after different treatments. Scale bar: 50 µm. (C) Intracellular ROS levels in TBHP‐pretreated AFCs measured by flow cytometry analysis and quantitative results. (D,E) Quantitative analysis of MDA and GSH levels in TBHP‐pretreated AFCs co‐cultured with QG, TMH and TMH/QG MN (with NIR irradiation). (F,G) Representative images and quantification of labile iron pool by FerroOrange staining and lipid peroxidation by C11‐BODIPY staining in TBHP‐pretreated AFCs co‐culture with QG, TMH and TMH/QG MN (with NIR irradiation). Scale bar: 20 µm. (H) Western blot analysis and corresponding quantitative statistics of extracellular matrix (COL1), remodeling markers (MMP3, MMP13), and ferroptosis‐related proteins (ACSL4, GPX4). The corresponding uncropped blot images are provided in Table S1. (I) Immunofluorescence staining and fluorescence intensity quantification of COL1 and MMP3 expression in TBHP‐pretreated AFCs co‐cultured with QG, TMH, and TMH/QG MN (with NIR irradiation). Scale bar: 20 µm. Data are presented as mean ± SD (n ≥ 3), and *p*‐values were calculated using one‐way ANOVA with Tukey's multiple comparisons test [(B) to (I)].

To verify the therapeutic impact of TMH/QG+NIR core‐shell microneedles on AFCs, we established a co‐culture system of AFCs with microneedle formulations under oxidative stress (Figure 5A). We first assessed the cytocompatibility of the microneedle formulations (shell‐layer TMH, core‐layer QG, and core‐shell TMH/QG) using live/dead staining after 1 and 3 days of co‐incubation with normal AFCs. Cell viability remained unaffected in all microneedle‐treated groups regardless of NIR irradiation, confirming they had excellent biocompatibility to support further in vitro and in vivo application (Figure S8A,C). Moreover, the bright‐field microscopy images revealed that no obvious changes in cell morphology or proliferation were observed in AF cells after 1 and 3 days of co‐culture with all types of microneedle materials, indicating good cytocompatibility of the microneedle formulations (Figure S8B). To model degenerative AFCs under oxidative stress, we used tert‐butyl hydroperoxide (TBHP) pretreated AFCs and evaluated the rescue effects of the microneedles via CCK‐8 assay. The results showed that shell‐layer TMH, core‐layer QG and core‐shell TMH/QG all enhanced cell viability under TBHP challenge, with the core‐shell TMH/QG group exhibiting the strongest protection, which increased cell viability by approximately 1.56‐fold over untreated TBHP‐induced AFCs (Figure S8D).

Based on the above results of the ROS‐resistance of microneedles, we further analyzed intracellular ROS levels using DCFH‐DA fluorescence staining. The staining images and their corresponding quantitative analyses revealed that TBHP stimulation markedly elevated ROS of AFCs, but the treatment with TMH+NIR, QG+NIR or TMH/QG+NIR substantially attenuated this increase (Figure 5B). Notably, the TMH/QG+NIR group reduced ROS levels by 77.17%, which restored them to near baseline levels observed in normal AFCs (Con group). Additionally, the results of flow cytometry corroborated these findings, indicating a statistically significant reduction in ROS following TMH/QG+NIR treatment (*p* < 0.0001, Figure 5C). Since persistent intracellular ROS would promote lipid peroxidation and exhaust endogenous antioxidants, we measured MDA and GSH levels of different samples. All NIR‐activated microneedle groups significantly lowered MDA content and elevated GSH levels in TBHP‐stimulated AFCs relative to untreated degenerative controls (Figure 5D,E).

Furthermore, fluorescence imaging with Liperfluo confirmed that NIR+microneedle treatments substantially attenuated lipid peroxide (LPO) accumulation (Figure 5G). Since Fe^2+^ catalyzes ROS production via the Fenton reaction, we next quantified intracellular Fe^2+^ levels using FerroOrange staining. Quantitative analysis demonstrated that TMH+NIR, QG+NIR and TMH/QG+NIR significantly reduced Fe^2+^ accumulation under TBHP‐induced oxidative stress (Figure 5F). Building on the above results, molecular analyses of RT‐qPCR and western blot further demonstrated that the treatment of TMH+NIR, QG+NIR and TMH/QG+NIR upregulated ferroptosis‐suppressor GPX4 and downregulated ferroptosis‐driver ACSL4 expression at mRNA and protein levels compared to those of the TBHP group (Figure 5H and Figure S8E), illustrating that the microneedles enhanced cellular resistance to ferroptosis under oxidative stress conditions.

Beyond mitigating ferroptosis, microneedle treatments also promoted ECM regeneration, as evidenced by increased collagen type 1 (COL1) expression and reduced levels of the matrix‐degrading enzymes matrix metalloproteinase 13 (MMP13) and matrix metalloproteinase 3 (MMP3) at both transcriptional and translational levels (Figure 5H, Figure S8E). Immunofluorescence (IF) staining with corresponding quantification further confirmed these coordinated changes of COL1 and MMP3 expression in all groups (Figure 5I). Across all of the above assays, the core‐shell TMH/QG microneedles combined with NIR irradiation produced more pronounced therapeutic effects than either single‐component formulation (TMH+NIR and QG+NIR). Collectively, these findings demonstrated that the combined treatment of TMH/QG+NIR effectively attenuated oxidative stress, suppressed ferroptosis, and restored matrix metabolic balance in degenerative AFCs under in vitro conditions.

### 2.5 TMH/QG+NIR Alleviates Ferroptotic Injury in Degenerative AFCs by Targeting ATF3

**FIGURE 6:**
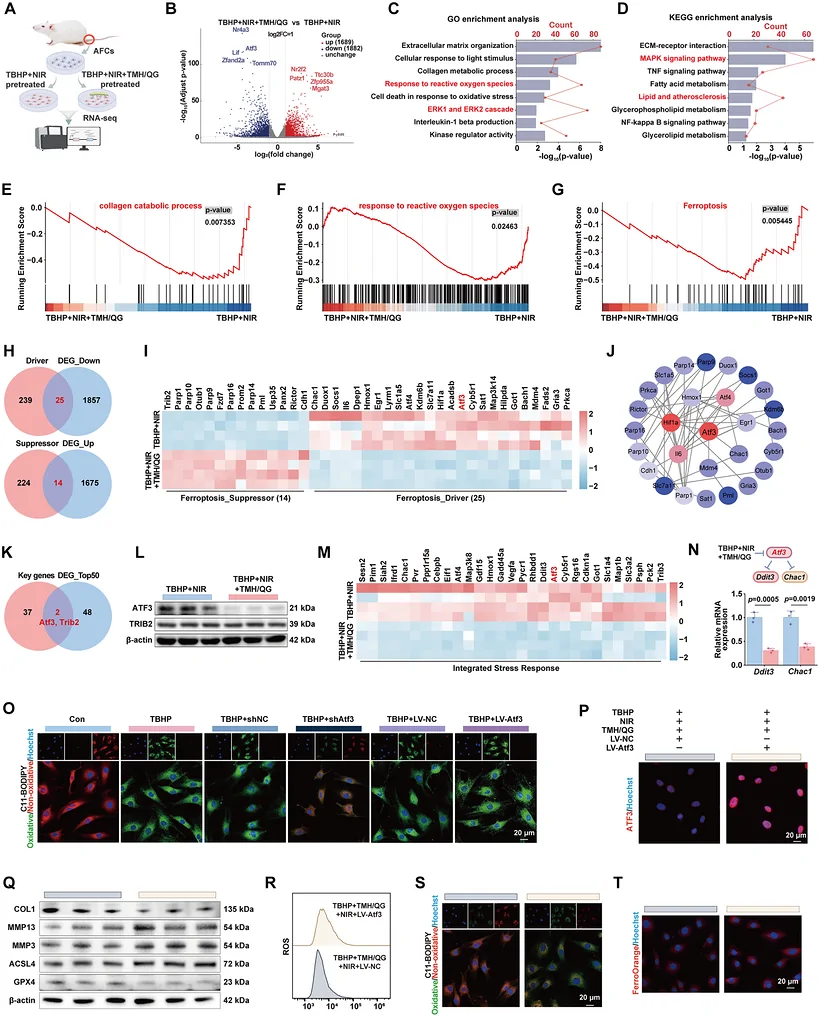
Transcriptomic profiling identifies ATF3 as a key regulator of TMH/QG+NIR‐mediated ferroptosis inhibition in degenerative AFCs. (A) RNA sequencing of AFCs treated with either TBHP+NIR or TBHP+NIR+TMH/QG. (B) Volcano plot identifying 3571 DEGs between TBHP+NIR and TBHP+NIR+TMH/QG groups. (C,D) Functional enrichment analysis of DEGs using GO biological processes and KEGG pathways. (E–G) GSEA plots demonstrating significant enrichment of processes related to extracellular matrix degradation, oxidative stress and ferroptosis. (H) 25 key genes identified from the intersection of ferroptosis driver genes and DEG_Down, 14 key genes identified from the intersection of ferroptosis suppressor genes and DEG_Up. (I) Cluster analysis of DEGs highlighting specific ferroptosis driver and suppressor genes. (J) PPI network of the top 28 hub genes with Atf3 ranked as the top hub gene. (K) 2 key genes (*Atf3* and *Trib2*) identified from the intersection of key genes and DEG_Top50. (L) Western blot analysis of ATF3 and TRIB2 protein expression levels in TBHP+NIR and TBHP+NIR+TMH/QG groups. The corresponding uncropped blot images are provided in Table S1. (M) Cluster analysis of DEGs showing the Integrated Stress Response between TBHP+NIR and TBHP+NIR+TMH/QG groups. (N) Relative mRNA expression levels of *Ddit3* and *Chac1*. (O) Representative images and quantitative analysis of C11‐BODIPY staining to assess lipid peroxidation in shAtf3 and LV‐Atf3 groups. Scale bar: 20 µm. (P) Immunofluorescence staining of ATF3 in the TBHP+TMH/QG+NIR+LV‐Atf3 group compared to the TBHP+TMH/QG+NIR+LV‐NC control group. Scale bar: 20 µm. (Q) Western blot analysis of ECM (COL1, MMP3, and MMP13) and ferroptosis (ACSL4 and GPX4) markers in TBHP+TMH/QG+NIR+LV‐Atf3 and TBHP+TMH/QG+NIR+LV‐NC groups. The corresponding uncropped blot images are provided in Table S1. (R) Flow cytometry analysis of intracellular ROS levels in TBHP+TMH/QG+NIR+LV‐Atf3 and TBHP+TMH/QG+NIR+LV‐NC groups. (S,T) C11‐BODIPY and FerroOrange staining images in TBHP+TMH/QG+NIR+LV‐Atf3 and TBHP+TMH/QG+NIR+LV‐NC groups. Scale bar: 20 µm. Data are presented as mean ± SD (n ≥ 3), and *p*‐values were calculated using two‐tailed Student's *t‐*test (N).

Because the TMH/QG+NIR microneedle system attenuated oxidative stress and ferroptosis‐associated injury in degenerative AFCs, we further investigated the underlying mechanism using RNA sequencing (Figure 6A). As shown in Figure 6B and Figure S9A, the comparative analysis between TBHP+NIR and TBHP+NIR+TMH/QG groups identified 3571 differentially expressed genes (DEGs), with 1882 downregulated and 1689 upregulated DEGs. Functional enrichment analysis using Gene Ontology (GO) enrichment and Kyoto Encyclopedia of Genes and Genomes (KEGG) pathway analyses revealed significant involvement of these genes in biological processes, including response to reactive oxygen species (ROS), ERK1 and ERK2 cascade (Figure 6C), MAPK signaling, and lipid and atherosclerosis (Figure 6D). Additionally, Gene Set Enrichment Analysis (GSEA) further confirmed enrichment in collagen catabolic process (p value = 0.007353), response to ROS (p value = 0.02463), macrophage activation (p value = 0.01254), collagen degradation (p value = 0.01357), ferroptosis (p value = 0.005445), cytokine activity (p value = 0.001709), MAPK signaling pathway (p value = 0.01544), and ERK1/ERK2 cascade (p value = 0.001626) (Figure 6E–G and Figure S9B,C). We next identified 39 ferroptosis‐related candidate genes, including 25 genes shared by ferroptosis drivers and downregulated DEGs and 14 genes shared by ferroptosis suppressors and upregulated DEGs (Figure 6H,I). Protein‐protein interaction (PPI) network analysis identified 28 hub nodes, among which Activating Transcription Factor 3 gene (*Atf3*) consistently appeared across multiple gene sets and ranked as a top hub candidate (Figure 6J). Intersection analysis between the top 50 DEGs (DEG_Top50) and 39 key genes further refined two core regulators: *Atf3* and *Trib2* (Figure 6K). The mRNA level of *Atf3* decreased by 22.34%, whereas the mRNA expression of *Trib2* showed no significant difference (Figure S9D). Meanwhile, western blot validation further confirmed that the protein expression level of ATF3 was significantly reduced following TMH/QG+NIR treatment compared to TBHP+NIR controls (p = 0.0010), but there was no alteration in the expression of TRIB2 between the above‐mentioned groups (Figure 6L and Figure S9E), leading to the selection of ATF3 for further studies. In addition, cluster analysis of DEGs further revealed distinct activation of the Integrated Stress Response between TBHP+NIR and TBHP+NIR+TMH/QG groups (Figure 6M), which contained *Atf3* and it‐mediated downstream factors (*Ddit3* and *Chac1*), and their expression levels also reduced by 69.39% (p = 0.0005) and 61.82% (p = 0.0019) after TMH/QG+NIR treatment (Figure 6N).

To functionally validate the role of ATF3 in degenerative AFCs, we generated stable AFC lines with either ATF3 knockdown (shAtf3) or overexpression (LV‐Atf3). The transduction efficiency was confirmed by western blot analysis, which showed that ATF3 protein levels in shAtf3_1, shAtf3_2 and shAtf3_3 lentiviral groups were reduced to 12.70%, 6.11%, and 10.61% of control levels, respectively, while the LV‐Atf3 group exhibited a 444.93% increase in ATF3 expression, confirming successful establishment of these genetic models (Figure S9F,G). Subsequently, we employed the C11‐BODIPY probe to assess cellular lipid peroxidation levels, in combination with the western blot of ferroptosis markers (GPX4 and ACSL4) to analyze the association between ATF3 expression and ferroptosis progression in AFCs. The shAtf3 group displayed significantly lower LPO levels relative to TBHP‐stimulated controls (*p* < 0.0001), along with a 40.18% decrease in ACSL4 and a 1.28‐fold increase in GPX4 protein expression (Figure 6O and Figure S9H,J). Conversely, the LV‐Atf3 group revealed markedly elevated LPO levels (p = 0.0011), accompanied by a 1.44‐fold increase in ACSL4 and a 60.19% reduction in GPX4 expression (Figure 6O and Figure S9I,J). These results indicate that ATF3 promotes lipid peroxidation and a ferroptosis‐associated molecular profile in degenerative AFCs.

To determine whether ATF3 mediates the therapeutic action of the TMH/QG+NIR microneedle system, we evaluated its effects in TBHP‐stimulated AF cells overexpressing ATF3 (LV‐Atf3). IF staining and quantitative analysis revealed that nuclear ATF3 expression remained elevated in the TBHP+TMH/QG+NIR+LV‐Atf3 group compared to the TBHP+TMH/QG+NIR+LV‐NC control group (Figure 6P and Figure S9K). Besides, flow cytometry and fluorescent staining assays of C11‐BODIPY and FerroOrange showed that TMH/QG+NIR treatment failed to reduce intracellular ROS, lipid peroxides and Fe^2+^ levels, respectively (Figure 6R–T and Figure S9L–P). Concurrently, the protein expression of ACSL4 increased again by 1.33‐fold, while GPX4 decreased by 29.91% (Figure 6Q and Figure S9L), indicating a reversal of the ferroptosis‐resistant effect. These results demonstrated that sustained ATF3 expression disrupted the therapeutic efficacy of TMH/QG+NIR, reactivating oxidative stress and ferroptosis in degenerative AFCs. Consistently, ECM homeostasis was also compromised under high ATF3 expression. Specifically, the elevation of the concentration of MDA (Figure S9M) was accompanied by decreased COL1 and increased MMP13 and MMP3 protein expression (Figure 6Q and Figure S9L) under the increased ATF3 expression in LV‐Atf3 AF cells. These results suggested that ATF3 overexpression abrogated the beneficial effects of the TMH/QG+NIR microneedle system, confirming ATF3 as a critical target through which this intervention alleviates oxidative damage, suppresses ferroptosis, and promotes matrix restoration.

### 2.6 TMH/QG+NIR Epigenetically Suppresses ATF3‐Driven Ferroptosis Through DNMT3A‐Mediated Promoter Methylation

To further investigate the mechanism by which the TMH/QG+NIR microneedle system targeted ATF3 to suppress ferroptosis of degenerative AFCs, we first assessed the stability of *Atf3* mRNA. AFCs in the TBHP+NIR and TBHP+NIR+TMH/QG groups were treated with 5 µg/mL of actinomycin D (ActD), a transcription inhibitor, and the gene expression degree of *Atf3* was detected after treating for 0, 0.5, 1, 2 and 4 h, respectively. The results revealed that there was no significant difference in *Atf3* mRNA expression between the TBHP+NIR+ActD and TBHP+NIR+TMH/QG+ActD groups, indicating that TMH/QG+NIR did not alter *Atf3* mRNA stability, suggesting transcriptional rather than post‐transcriptional regulation (Figure S10A).

**FIGURE 7:**
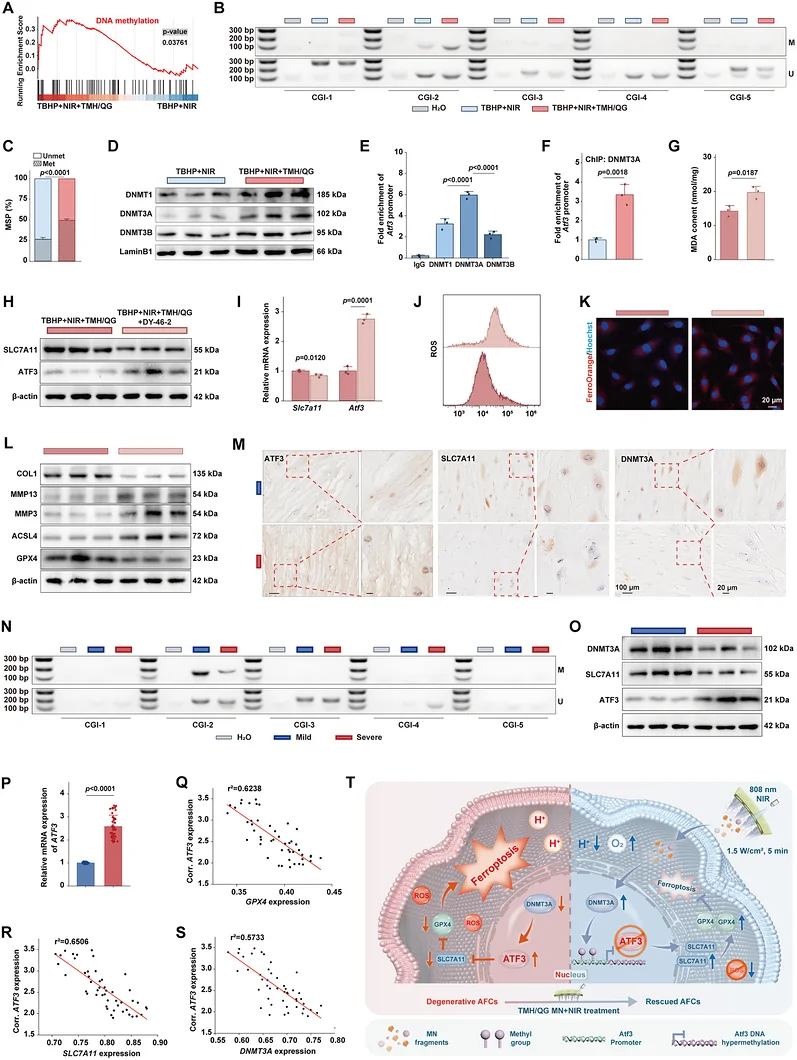
DNMT3A‐mediated ATF3 promoter methylation underlies the anti‐ferroptotic effect of TMH/QG+NIR in degenerative AFCs. (A) GSEA plots identify significant enrichment of DEGs in the DNA methylation pathway. (B,C) Methylation‐specific PCR and corresponding quantification of the *Atf3* promoter methylation level in degenerative AFCs treated with the TMH/QG+NIR microneedle system. The corresponding uncropped gel images are provided in Table S1. (D) Western blot analysis of DNA methyltransferases (DNMT1, DNMT3A, and DNMT3B) in the TBHP+NIR and TBHP+NIR+TMH/QG groups. The corresponding uncropped blot images are provided in Table S1. (E) ChIP‐qPCR analysis of DNMT1, DNMT3A, and DNMT3B enrichment at the *Atf3* promoter region. (F) ChIP‐qPCR analysis specifically quantifying DNMT3A enrichment at the *Atf3* promoter. (G) Measurement of MDA content. (H) Western blot analysis of SLC7A11 and ATF3 protein levels in the TBHP+NIR+TMH/QG group with or without DY‐46‐2. The corresponding uncropped blot images are provided in Table S1. (I) Relative mRNA expression level of *Slc7a11* and *Atf3* in the TBHP+NIR+TMH/QG group with or without the DNMT3A inhibitor DY‐46‐2. (J) Flow cytometry analysis of intracellular ROS levels. (K) Representative FerroOrange staining images in the TBHP+NIR+TMH/QG group with or without DY‐46‐2. Scale bar: 20 µm. (L) Western blot analysis of key ferroptosis (ACSL4 and GPX4) and extracellular matrix (COL1, MMP3 and MMP13) markers in the TBHP+NIR+TMH/QG group with or without DY‐46‐2. The corresponding uncropped blot images are provided in Table S1. (M) IHC staining of ATF3, SLC7A11 and DNMT3A expression in human disc tissues from mild and severe degeneration groups. Scale bar: 20 µm. (N) MSP of the *ATF3* promoter methylation level in human mild and severe degeneration groups. The corresponding uncropped gel images are provided in Table S1. (O) Western blot analysis of ATF3, SLC7A11 and DNMT3A protein expression in human disc tissues from mild and severe degeneration groups. The corresponding uncropped blot images are provided in Table S1. (P) Relative mRNA expression of *ATF3* in human mild and severe degeneration groups. (Q–S) Correlation analysis of relative *ATF3* expression in AF tissue and relative *GPX4*, *SLC7A11* and *DNMT3A* expression. (T) Schematic model illustrating the proposed mechanism. Data are presented as mean ± SD (n ≥ 3), and *p*‐values were calculated using two‐tailed Student's *t*‐test [(C), (F), (G), (I) and (P)] and one‐way ANOVA with Tukey's multiple comparisons test (E).

Due to the result of GSEA analysis in Figure 7A and Figure S10B, which indicated enrichment of DNA repair and DNA methylation‐related terms in the TBHP+NIR+TMH/QG group, we hypothesized that methylation modification might account for ATF3 downregulation. To investigate the association between *Atf3* inhibition and DNA methylation, we analyzed the rat *Atf3* promoter region using the online tool MetPrimer and identified five high‐density CpG islands (CGIs) in the *Atf3* promoter, suggesting susceptibility to methylation‐mediated regulation (Figure S10C).

Based on it, we designed and synthesized methylation‐specific primers (Table S2) for each CpG island, and employed the methylation‐specific PCR to evaluate the DNA methylation level of the *Atf3* promoter regions in degenerative AFCs co‐cultured with TMH/QG+NIR microneedle system. As shown in Figure 7B,C, the results indicated that *Atf3* was particularly sensitive to DNA methylation modification in the CGI‐2 region, which revealed a pronounced increase in methylation level from 27% in controls to 49% following TMH/QG+NIR microneedle treatment. To explore the regulatory role of DNA methylation writers (DNMT1, DNA methyltransferase 1, DNMT3A, DNA methyltransferase 3A and DNMT3B, DNA methyltransferase 3B) in *Atf3* gene methylation, we analyzed the expression levels of DNMT1, DNMT3A and DNMT3B using Western blotting and Chromatin Immunoprecipitation‐quantitative PCR (ChIP‐qPCR). The results in Figure 7D and Figure S10D–F showed that TMH/QG+NIR treatment significantly enhanced DNMT3A expression (*p* < 0.0001), and its enrichment level in the *Atf3* promoter region showed a similar trend (Figure 7E,F). These results suggested increased DNMT3A‐associated methylation at the CGI‐2 region of the Atf3 promoter.

To verify this hypothesis, we inhibited the activity of DNMT3A in the degenerative AFCs with the DNA methyltransferase inhibitor (DY‐46‐2) during the TMH/QG+NIR therapeutic progression. As shown in Figure 7H,I and Figure S10G,H, this microneedle treatment increased Atf3 mRNA and protein levels by 2.74‐fold and 2.95‐fold, and decreased the mRNA and protein levels of Solute Carrier Family 7 Member 11 (SLC7A11) by 28.5% and 15.68%, respectively. Notably, the immunofluorescence intensity of SLC7A11 decreased by 38.85% upon DY‐46‐2 intervention. These results indicated that pharmacological inhibition of DNMT3A activity by DY‐46‐2 reduced Atf3 promoter methylation and restored ATF3 expression, accompanied by decreased expression of SLC7A11. Additionally, the DY‐46‐2 treatment group also reversed the protective effects of the TMH/QG+NIR microneedle system, leading to elevated ROS (Figure 7J and Figure S10I), Fe^2+^ (Figure 7K and Figure S10J), and MDA (Figure 7G) levels significantly, accompanied by downregulation of GPX4 and upregulation of ACSL4 (Figure 7L and Figure S10K), which demonstrated that the introduction of DY‐46‐2 would re‐facilitate the ferroptosis process. In addition, the TMH/QG+NIR microneedle system‐induced reestablishment of matrix homeostasis was also impaired under the DY‐46‐2 treatment, as shown by reduced protein expression of COL1 by 72.19%, and elevated MMP3 and MMP13 by 4.58‐fold and 2.55‐fold, respectively (Figure 7L and Figure S10K). The above results confirmed that inhibition of DNMT3A activity partially reversed Atf3 repression, reduced SLC7A11 expression, and restored ferroptosis‐associated oxidative injury, indicating that the DNMT3A‐Atf3 methylation axis contributes to the anti‐ferroptotic action of TMH/QG+NIR (Figure 7T).

Subsequently, we further validated this mechanism in human AF tissues. The results of the western blot, IHC staining, and RT‐qPCR showed that the severe degeneration samples exhibited a 2.7‐fold increase in ATF3 protein with a decrease of 52.28% and 52.95% in DNMT3A and SLC7A11 protein, a higher proportion of immunopositive cells in the ATF3 area (70.6% vs. 48.8%) with a lower in the DNMT3A and SLC7A11 areas, and a 2.58‐fold rise in mRNA levels of *ATF3* compared to mild degeneration samples with a decrease of 28.95% and 22.47% in *DNMT3A* and *SLC7A11* mRNA levels (Figure 7M,O,P and Figure S10N–R). The online tool MetPrimer was used to analyze the human *ATF3* gene promoter region, which also identified five high‐density CpG islands (CGI‐1 to CGI‐5), indicating that the human *ATF3* gene was susceptible to regulation by DNA methylation modification as well (Figure S10L). Methylation‐specific primers were designed for each CpG island (Table S3) and employed the methylation‐specific PCR to detect the DNA methylation status of *ATF3* in the mild and severe groups of human samples. As shown in Figure 7N and Figure S10M, *ATF3* gene was particularly sensitive to methylation modification in the CGI‐2 region, with the methylation level in the severe degeneration group (26%) being significantly lower than that in the mild degeneration group (48%). In addition, correlation analysis revealed that *ATF3* expression was negatively correlated with *GPX4*, *SLC7A11* and *DNMT3A* expression in human AF tissue, which was consistent with the aforementioned in vitro experimental results to prove our hypothesis (Figure 7Q–S).

### 2.7 TMH/QG+NIR Preserves AF Structure and Disc Biomechanical Function In Vivo

**FIGURE 8:**
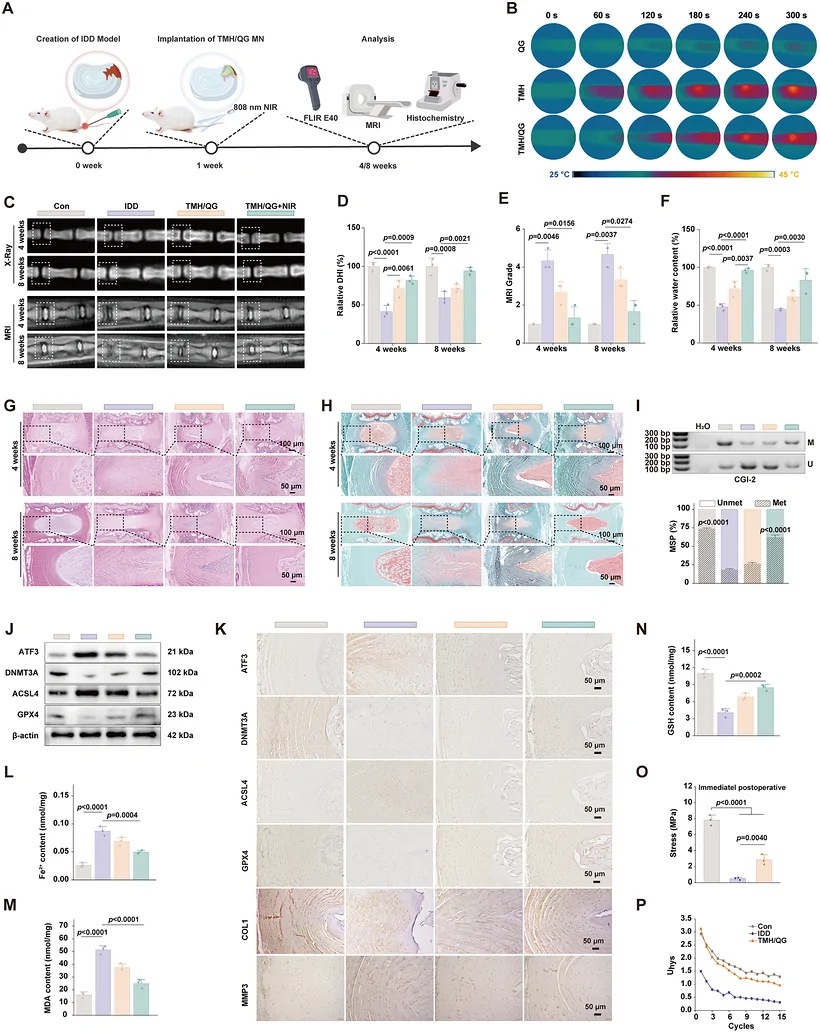
Preclinical evaluation of the TMH/QG+NIR strategy for AF repair. (A) Schematic illustration of the therapeutic strategy for rat tail puncture‐induced IDD using TMH/QG MN under NIR irradiation. (B) Infrared thermographic images of rat tails treated with QG, TMH and TMH/QG MNs under NIR irradiation. (C) Representative X‐ray and T2‐weighted MRI images of rat tails from the indicated treatment groups at 4‐ and 8‐weeks post‐treatment. (D–F) Quantitative assessment of disc degeneration: DHI, MRI grading scores and disc water content at 4 and 8 weeks (n = 5). (G,H) Histological analysis of AF tissues at 4 and 8 weeks. Representative H&E and SO/FG staining images. (I) Methylation‐specific PCR analysis and quantification of the CpG island CGI‐2 region in the rat *Atf3* promoter. The corresponding uncropped gel images are provided in Table S1. (J) Western blot analysis of ATF3, DNMT3A and ferroptosis markers (ACSL4 and GPX4) in disc tissues. The corresponding uncropped blot images are provided in Table S1. (K) Immunohistochemical staining of ATF3, DNMT3A, ACSL4, GPX4, COL1 and MMP3 expression in AF tissues. (L–N) Biochemical assays of ferroptosis‐related factors in disc tissues: Fe^2+^ content, MDA level and GSH level at 8 weeks post‐treatment. (O) Compressive modulus of IVDs from the Con, IDD and TMH/QG+NIR groups. (P) Energy dissipation efficiency (U_hys_) of IVDs from Con, IDD and TMH/QG+NIR groups. Data are presented as mean ± SD (n ≥ 3), and *p*‐values were calculated using one‐way ANOVA with Tukey's multiple comparisons test [(D), (F), (I), and (L) to (O)] and Kruskal‐Wallis test with Dunn's multiple comparisons test (E).

Before the in vivo assessment of the TMH/QG+NIR system, we evaluated its biosafety by histological examination of major organs after implantation into the Sprague‐Dawley (SD) rats' IVD for 8 weeks. Hematoxylin and eosin (H&E) staining images and corresponding semi‐quantitative scoring of inflammatory infiltration [38, 39, 40, 41, 42] revealed that there were no notable pathological alterations or enhanced inflammatory infiltration in any major organs from the TMH/QG or TMH/QG+NIR groups compared with normal controls, which confirmed satisfactory biocompatibility of TMH/QG or TMH/QG+NIR for further preclinical evaluation (Figure S11A). Meanwhile, as shown in Figure S11B, no significant alterations were observed in the complete blood count (CBC) parameters, including white blood cells (WBCs), hemoglobin (HGB), red blood cells (RBCs), mean corpuscular volume (MCV), mean corpuscular hemoglobin (MCH), and platelets (PLT) in SD rats with AF defects following discectomy in the TMH/QG‐treated groups. These findings further confirmed that TMH/QG and TMH/QG+NIR are suitable for subsequent evaluation of in vivo therapeutic efficacy. To ensure the efficiency of TMH/QG+NIR for degenerative AF in IDD, we subsequently measured the photothermal effect degeneration performance in vivo. As shown in Figure 8B and Figure S11C, the images of infrared thermography revealed that the TMH/QG+NIR group addressed a mild photothermal therapy, with the local temperature of approximately 40°C within 300 s, which was a range conducive to AF tissue repair. Additionally, in vivo degradations of microneedles were tracked over 21 days, and the relevant degradation ratios were 98.02% for core‐layer QG MN, 89.75% for shell‐layer TMH MN, 75.83% for core‐shell TMH/QG MN, and 64.85% for core‐shell TMH/QG MN with NIR irradiation treatment, which exhibited a suitable degradation rate to avoid a low therapeutic effect caused by rapid degradation, as well as the restriction of AF regeneration due to long‐term residence in the tissue (Figure S11D).

To assess the preclinical therapeutic efficiency of core‐shell TMH/QG MN with/without NIR irradiation treatment of degenerative AF in IDD progression, we established the SD rat tail puncture‐induced IDD model, and the interventions of TMH/QG MN were administered at post‐surgery for 1 week (Figure 8A). Radiological examination by X‐ray and magnetic resonance imaging (MRI) after 4 and 8 weeks of treatment indicated moderate structural recovery in both TMH/QG and TMH/QG+NIR groups relative to untreated IDD group (Figure 8C). Specifically, the TMH/QG+NIR group exhibited the most pronounced restorative effect, with the disc height index (DHI) improving from 59.47% to 94.16% (Figure 8D), alongside a 64.29% reduction in MRI‐based degeneration grade (Figure 8E) and a 1.87‐fold increase in water content after therapy for 8 weeks (Figure 8F). These morphological and compositional metrics in the TMH/QG+NIR group approached those of healthy control IVD (Con group).

In addition, histological evaluation through H&E and safranin O/fast green (SO/FG) staining further supported the protective role of TMH/QG+NIR against microstructural disintegration. The results of Figure 8G,H indicated that TMH/QG+NIR could preserve AF organization and NP integrity, and the treated IVD closely mirrored the architecture of the healthy tissue in the Con group. The relevant quantitative histological analysis in Figure S11E corroborated the above‐mentioned observations, which performed a 55.91% decrease in the histological grade of the TMH/QG+NIR group compared to the IDD group, demonstrating the structural benefits of TMH/QG+NIR intervention in mitigating IDD in vivo.

Based on evidence in vitro that TMH/QG+NIR treatment could suppress ATF3 transcription by enhancing its DNA methylation, we further verified this epigenetic mechanism in the SD rat IDD model. As shown in Figure 8J,K and Figure S11F,G, the results of RT‐qPCR, western blot, and IHC staining analyses revealed that TMH/QG+NIR treatment significantly downregulated ATF3 expression both in the mRNA and protein levels, with IHC staining intensity and distribution closely resembling those in healthy IVD in Con group. Additionally, methylation‐specific PCR targeting the CpG island CGI‐2 region of the SD rat *Atf3* gene showed methylation levels of 74% in normal IVD (Con group), 18% in degenerated IVD (IDD group), 26% in TMH/QG‐treated IVD (TMH/QG group), and 62% in TMH/QG+NIR‐treated IVD (TMH/QG+NIR group), indicating the methylation level of the *Atf3* gene in IVD of the TMH/QG+NIR group was the closest to the normal IVD level (Figure 8I). Subsequently, we further evaluated the expression of DNA methyltransferase (DNMT3A), which catalyzes DNA methylation. The results of RT‐qPCR, western blot, and IHC staining consistently demonstrated that the mRNA and protein levels of DNMT3A were significantly elevated in AF tissues from both TMH/QG and TMH/QG+NIR groups compared to degenerated controls, with the TMH/QG+NIR group approaching levels observed in healthy IVD (Figure 8J,K and Figure S11F,G–I). The above results indicated that TMH/QG+NIR promoted DNMT3A expression to facilitate its binding to the *Atf3* promoter, increasing local DNA methylation, and ultimately inhibiting *Atf3* transcription in degenerative AF during IDD progression.

Furthermore, we also assessed oxidative stress levels in AF tissues by biochemical assays. Measurement of GSH and MDA contents revealed that both TMH/QG and TMH/QG+NIR treatments significantly increased GSH while reducing MDA accumulation in the AF relative to untreated degenerated samples (IDD group). Besides, the TMH/QG+NIR group exhibited values closest to those of healthy controls in the Con group, indicating effective mitigation of oxidative damage (Figure 8M,N). Then, we further evaluated ferroptosis activity by quantifying Fe^2+^ levels and measuring the expression of the ferroptosis driver ACSL4 and suppressor GPX4. The results demonstrated that the TMH/QG+NIR group showed markedly reduced Fe^2+^ content and ACSL4 expression, while enhancing GPX4 levels at both mRNA and protein levels, which closely resemble the profile of normal AF tissues (Figure 8J–L and Figure S11F,G,J,K). These results illustrated that the TMH/QG+NIR treatment performed robust inhibition of ferroptosis and ferrous ion accumulation in degenerative AF during IDD progression. Furthermore, the analysis of IHC staining of ECM homeostasis markers indicated TMH/QG+NIR treatment could elevate COL1 and diminish MMP3 expression in degenerative AF tissues (Figure 8K and Figure S11L,M), while these therapeutic effects persisted throughout the 8‐week post‐surgery, reflecting that TMH/QG+NIR treatment could effectively restore ECM homeostasis in vivo.

To further evaluate the mechanical functional recovery induced by TMH/QG+NIR treatment, we examined the mechanical properties of IVD from each group using a multi‐scale in situ mechanical testing system (IBTC‐300). The compressive stress‐strain curves and their corresponding quantitative analyses obtained from Con, IDD and TMH/QG+NIR groups indicated that TMH/QG+NIR treatment restored the compressive modulus from 0.48 MPa in degenerated IVD (IDD group) to 2.84 MPa, approaching the 7.81 MPa observed in healthy controls (Con group) (Figure 8O and Figure S12A). In addition to compressive performance, the fatigue resistance of IVDs is also essential for adapting to complex mechanical forces in the microenvironment. We further detected the fatigue resistance of IVDs after different treatments through 15‐cycle compression tests. As shown in Figure 8P and Figure S12C, the results revealed enhanced fatigue resistance in the TMH/QG+NIR group, which exhibited a maximum compressive stress 2.21‐fold higher than that of the IDD group. Meanwhile, energy dissipation in this treatment group closely matched that of control discs, with dissipation efficiency increasing from 31% in degenerated IVD to 45.5%, which was near the 51.4% efficiency measured in normal IVDs (Figure S12B). These mechanical assessments all suggested that TMH/QG+NIR treatment effectively restored structural support and improved functional resilience in degenerated IVD.

## 3 Discussion

IDD is a prevalent spinal disorder and serves as the primary etiological factor underlying LBP, which profoundly impairs patients’ quality of life and work capacity [43]. The AF forms a fibrous connective tissue barrier around the NP as a key component of the IVD, providing structural support while maintaining biomechanical stability through the ordered layering of collagen fibers [44, 45, 46]. Once the AF ruptures during degeneration, it would disrupt local biomechanical balance, and the NP may herniate and compress nerve roots to cause the LBP and even limb dysfunction in severe cases [4, 35, 47].

To elucidate the core pathological mechanisms underlying AF injury, we performed bioinformatics analysis on single‐cell RNA sequencing data derived from AF tissues of patients with mild (Pfirrmann grade II‐III) and severe (Pfirrmann grade IV‐V) IDD and identified 6284 DEGs in total. Functional enrichment analysis demonstrated that these genes were significantly enriched in pathways such as fatty acid metabolism, ferroptosis regulation, ROS scavenging, lipid oxidation and iron ion transport, all of which are closely associated with the key pathological processes of IDD. Experimental validation in human degenerative IVD tissues revealed two characteristic alterations in severe degeneration, namely ferroptosis activation indicated by increased ACSL4 expression, elevated Fe^2+^/lipid peroxidation levels, decreased GPX4 levels, and redox homeostasis imbalance reflected by increased malondialdehyde levels and decreased glutathione levels. These findings support redox imbalance and ferroptosis activation as prominent pathological features associated with advanced AF degeneration, and that the two processes are tightly interrelated. Oxidative stress induces ferroptosis by promoting iron release and activating the Fenton reaction, whereas ferroptosis‐driven cell death causes massive release of Fe^2+^ and ROS, which further exacerbates oxidative stress. This vicious cycle not only accelerates AFC death and ECM degradation but also disrupts IVD microenvironment homeostasis, ultimately driving the progression of degeneration. These observations indicate that a more comprehensive therapeutic strategy is needed to intervene in the interaction between oxidative stress and ferroptosis.

Since the AF has limited self‐repair capacity, and the healing process after rupture is extremely slow [48, 49]. We developed a shell‐core TMH/QG MN system engineered to deliver coordinated mechanical support, antioxidative action and programmable photothermal therapy. Mechanical characterization confirmed the system's robustness for clinical application with a penetration force of approximately 6.00 N per needle, a value that significantly exceeds the threshold required for reliable AF insertion. The complementary mechanical properties of the shell and core layers provide high rigidity and exceptional ductility, respectively, which ensure structural integrity under the dynamic loading conditions of the intervertebral disc. The therapeutic value of TMH/QG MN is not simply derived from functional integration, but from its ability to translate the acidic degenerative AF microenvironment into a staged repair sequence. In the shell layer, TA@MgO_2_ nanobots respond to pathological H^+^ by consuming protons, generating O_2_, and enhancing redox buffering, thereby creating a permissive niche for subsequent QUE‐mediated ferroptosis suppression. The formation of metal‐phenolic networks with tannic acid does not induce a phase transformation or lattice distortion in the MgO_2_ core, thereby preserving its crystallographic structure and retaining its self‐propulsion capability in acidic microenvironments. Upon exposure to acidic conditions, these TA@MgO_2_ nanobots asymmetrically release oxygen bubbles to initiate self‐propulsion while consuming protons to raise the local pH, thereby ameliorating both hypoxia and acidosis in deeply degenerated AF tissue. Simultaneously, TA@MgO_2_ nanobots exhibit enzyme‐mimetic antioxidant behavior that concurrently quenches stable free radicals, scavenges superoxide anions through superoxide dismutase‐like activity, and efficiently decomposes hydrogen peroxide, disrupting the reactive oxygen species cascade at multiple nodes. Under NIR irradiation, TA@MgO_2_ nanobots further enable programmable photothermal amplification that delivers on‐demand and pulsatile antioxidant boosting, which significantly enhances reactive oxygen species decomposition and provides mild photothermal therapy in a precisely controllable manner. The shell‐core TMH/QG MN system thus embodies an integrated therapeutic strategy: its mechanically robust architecture ensures reliable implantation and retention, while the microenvironment‐responsive self‐propelled TA@MgO_2_ nanobots enable coordinated antioxidant and programmable photothermal functions that collectively remodel the pathological AF microenvironment. By simultaneously alleviating acidic accumulation, neutralizing oxidative stress, and applying mild phototherapy, the system establishes a conducive regenerative niche for structural and functional restoration of degenerative AF tissue.

Beyond the innovative material design, our work unveils a novel epigenetic circuit governing ferroptosis in AF cells. RNA‐seq‐based pathway analysis was used to prioritize transcriptional regulators associated with the anti‐ferroptotic effect of TMH/QG+NIR. Among the candidate ferroptosis‐related hub genes, ATF3 showed the most consistent treatment‐responsive reduction at both transcriptomic and protein levels. Functional gain‐ and loss‐of‐function experiments further demonstrated that ATF3 is sufficient to aggravate, and partly required to sustain, lipid peroxidation and ferroptosis in stressed AFCs. Consistently, ATF3 expression was upregulated in tissue samples from IDD patients, indicating its potential role in the progression of IDD. Previous studies have indicated that epigenetic modification is a key genetic regulatory mechanism influencing aging and age‐related diseases, [50, 51] among which DNA methylation is a critical factor accelerating age‐related degenerative processes [52, 53]. Therefore, considering the regulatory role of epigenetic modification in IDD, we further investigated the underlying mechanism of TMH/QG+NIR on ATF3. It was found that TMH/QG+NIR does not exert its effects by altering *Atf3* mRNA stability, excluding a post‐transcriptional regulatory mechanism, but rather inhibits ATF3 transcription by upregulating its methylation level. Analysis using the MetPrimer online tool revealed the presence of 5 high‐density CGIs in the *Atf3* promoter region; MSP confirmed that TMH/QG+NIR could significantly increase the methylation level of the CGI‐2 region (from 27% to 49%). DNMT3A was identified as a key upstream regulatory factor. TMH/QG+NIR can upregulate DNMT3A expression and promote its binding to the *Atf3* promoter, thereby mediating ATF3 methylation and transcriptional inhibition. In addition, ATF3 has been reported to function as a negative regulator of SLC7A11, thereby modulating cellular redox homeostasis [54, 56]. Downregulation of ATF3 may relieve its transcriptional repression on SLC7A11, leading to enhanced cystine uptake via system Xc^−^, increased intracellular cysteine availability, and subsequently elevated GSH biosynthesis [55, 56, 57]. Inhibition of DNMT3A with DY‐46‐2 abrogated the downregulatory effect of TMH/QG+NIR on ATF3, which was associated with the downregulation of SLC7A11, suppressing cystine import and GSH synthesis, thereby reactivating ferroptosis. Consistent with the in vitro mechanism, TMH/QG+NIR restored Atf3 promoter methylation and normalized DNMT3A/ATF3/SLC7A11‐associated ferroptosis markers to promote AF repair in vivo. Our findings thus position the DNMT3A‐ATF3 methylation axis as a critical regulatory pathway and a promising therapeutic target for IDD [58].

Despite the promising therapeutic outcomes demonstrated by our core‐shell TMH/QG+NIR microneedle system, several limitations warrant acknowledgment and should be addressed in future investigations. First, while we have established that the microneedle system upregulates DNMT3A to enhance *Atf3* promoter methylation and suppress ferroptosis, the precise upstream signaling pathways responsible for DNMT3A activation remain incompletely characterized. Specifically, the involvement of major signaling cascades such as MAPK, PI3K/AKT, and other redox‐sensitive pathways in mediating the microneedle‐induced epigenetic modifications requires further elucidation. Second, our study primarily utilized rodent models and in vitro cell cultures, which may not fully recapitulate the complex pathophysiological environment of human IDD. Although we validated key findings using human AF tissues from patients with varying degrees of IDD, the therapeutic efficacy and safety profile require validation in larger animal models with anatomical and biomechanical properties more closely resembling human spinal segments. Third, the long‐term biosafety profile beyond the 8‐week observation period necessitates thorough investigation, particularly regarding potential accumulation of degradation products from the microneedle components and their systemic effects following administration. Finally, because NIR penetration is limited in deep human lumbar discs, future translation of this strategy may require intraoperative, endoscopic, or image‐guided local irradiation rather than simple transcutaneous illumination. Addressing these limitations through multidisciplinary approaches will be crucial for advancing this innovative microneedle system toward clinical application for IDD treatment.

## 4 Conclusion

In conclusion, this study demonstrates that redox imbalance and ferroptosis activation represent fundamental pathological mechanisms underlying IDD progression, with the DNMT3A‐ATF3 methylation axis serving as a critical regulatory pathway in this degenerative process. Given that, we developed an acid‐responsive nanobot‐integrated core‐shell microneedle system for staged annulus fibrosus repair. The shell layer released TA@MgO_2_ metal‐phenolic nanobots that consumed pathological H^+^, generated O_2_ and provided NIR‐amplified redox buffering, thereby normalizing the early degenerative microenvironment. The core layer enabled sustained quercetin release to reinforce anti‐ferroptotic and matrix‐preserving effects. Mechanistically, TMH/QG+NIR suppressed ferroptosis in AF cells by epigenetically silencing ATF3 through DNMT3A‐dependent promoter methylation, thereby relieving ATF3‐mediated repression of SLC7A11 and restoring GPX4‐dependent antioxidant defense. In a puncture‐induced degeneration model, this system alleviated ferroptotic injury, improved AF integrity, preserved disc height and enhanced biomechanical resilience. These findings identify the DNMT3A‐ATF3‐SLC7A11 axis as a therapeutically tractable ferroptosis‐regulatory pathway and establish a staged microenvironment‐reprogramming strategy for AF repair.

## 5 Experimental Section

### 5.1 Ethical Statement

This study was conducted in accordance with the Declaration of Helsinki and was approved by the Ethics Committee of Xinqiao Hospital (Approval No. 2023‐YD184‐01), covering the collection of human tissues and use of associated clinical data. All experimental procedures involving animals were performed in strict accordance with institutional and national ethical guidelines and were reviewed and approved by the Experimental Animal Ethics Committee of the Third Military Medical University (Chongqing, China) (Approval No. AMUWEC20255389).

### 5.2 Synthesis and Characterization of Metal‐Phenolic Network

2 g of MgO were mixed with 30 mL 30% H_2_O_2_ solution under continuous magnetic stirring overnight. The resulting suspension was centrifuged at 13 000 × g for 5 min to collect the precipitate. The final MgO_2_ product was washed twice with absolute ethanol and then dispersed in absolute ethanol for further use. 10 mg of tannic acid (TA) was dissolved in 100 mL of distilled water (ddH_2_O), and MgO_2_ was dispersed in anhydrous ethanol (20 mg/mL), respectively. Subsequently, the TA solution and MgO_2_ dispersion were mixed at predetermined molar ratios in an ice bath. The reaction mixture was centrifuged at 13 000 × g for 10 min to remove the supernatant. The precipitate was washed three times with ddH_2_O and then dried in a vacuum oven at 40°C for 12 h to yield TA@MgO_2_ metal‐phenolic network (MPN).

Transmission electron microscopic (TEM) imaging was carried out on a JEOL JEM‐2010 TEM (acceleration voltage at 200 kV) to observe the morphology of MgO_2_ and TA@MgO_2_. X‐ray diffraction analysis (XRD) was utilized to detect the crystalline condition in MgO_2_ and TA@MgO_2_. The samples were placed on an Eluerian 1/4 cradle and diffraction data were collected using 40 kV, 40 mA Cu Kα radiation over a 2θ range from 10° to 80°. The surface chemical composition and ionic valences of MgO_2_ and TA@MgO_2_ were detected by X‐ray photoelectron spectroscopy (XPS) on an ESCALAB250Xi system (Thermo Scientific, USA). Zeta potential and dynamic light scattering (DLS) analysis of TA, MgO_2_ and TA@MgO_2_ were performed by a Malvern Zetasizer (Nano ZS, Malvern, UK).

To evaluate the photothermal performance of TA@MgO_2_, we prepared the TA@MgO_2_ with different concentrations and exposed it to continuous infrared irradiation with different powers for 5 min, while recording the temperature value every 30 s. Cycling photothermal stability was assessed using 1 mL of 1 mg/mL TA@MgO_2_ under high‐power irradiation (1.5 W/cm^2^), which underwent 5 heating‐cooling cycles. Thermal images of the 1 mg/mL TA@MgO_2_ under 1.5 W/cm^2^ irradiation were acquired at 60 s intervals by a FLIR E40 thermal camera and processed using FLIR Tools software to analyze temperature distributions.

### 5.3 Synthesis and Characterization of Core‐Shell Microneedles

For the synthesis of carboxymethyl‐β‐cyclodextrin (CM‐β‐CD), β‐cyclodextrin (10 g) and sodium hydroxide (9.3 g) were dissolved in 37 mL of ddH_2_O at 50°C. A chloroacetic acid solution (27 mL, 16.3% w/v) was then added dropwise, and neutralized to pH 5–6 using hydrochloric acid, and the CM‐β‐CD product was precipitated by adding methanol. Subsequently, GelMA (300 mg) was dissolved in 3 mL of DPBS (pH 7.4) at 80°C, and CM‐β‐CD (400 mg) was activated in 3 mL of MES buffer (pH 6.0) using EDC and NHS for 30 min. The activated CM‐β‐CD solution was reacted with the GelMA solution under alkaline conditions (pH 8–9) at 40°C for 12 h. The product was purified by dialysis (MWCO: 12–14 kDa) for 72 h and lyophilized to obtain the GelMA‐β‐CD. To encapsulate quercetin (QUE) using host‐guest interactions, GelMA‐β‐CD (2 g) was dissolved in 10 mL of DPBS (20% w/v), and the QUE solution (5 mg/mL) was added and stirred at 300 rpm and 37°C for 30 min. Upon completion of the reaction, the inclusion complex solution was transferred into a dialysis bag with a molecular weight cutoff (MWCO) of 12–14 kDa and dialyzed for 3 days to remove unencapsulated QUE. Finally, GelMA‐β‐CD@QUE was obtained via vacuum freeze‐drying. The above‐mentioned intermediate and final products were characterized by Fourier transform infrared spectroscopy (FTIR) and ^1^H nuclear magnetic resonance (^1^H NMR). Drug‐loading efficiency was determined quantitatively by UV–vis spectrophotometry.

To synthesize adipic dihydrazide (ADH) modified hyaluronic acid (HA), 1 g of HA was dissolved in 100 mL of ddH_2_O, then EDC (2.3 g), NHS (1.3 g) and ADH (4 g) were added to the solution (pH 6.8) for reacting at room temperature for 6 h. The resulting solution was dialyzed (MWCO: 800 Da) for 3 d to obtain hydrazide‐modified HA (HA‐ADH). 1 g of HA‐ADH was re‐dissolved in 100 mL of ddH_2_O, while 1 g of HCA was dissolved in a 50% ethanol solution for 1 h to activate the carboxyl groups by adding 2 g of EDC and 1.3 g of NHS. The activated HCA solution was then introduced into the HA‐ADH solution under a nitrogen atmosphere with stirring at 300 rpm and 100°C for 24 h. The product was subjected to acidic dialysis (pH 4.0) for 72 h and lyophilization to obtain HA‐ADH‐HCA. The chemical structures of HA‐ADH and HA‐ADH‐HCA were also characterized by FTIR and ^1^H NMR spectroscopy.

2 g of TA@MgO_2_ and 18 g of HA‐ADH‐HCA were added to 100 mL ddH_2_O to stir for 24 h, and the mixture was cast into the microneedle mold for 3 cycles of vacuum injection to eliminate internal and tip‐trapped bubbles, followed by curing in a constant‐temperature drying oven at 37°C for at least 24 h to form TA@MgO_2_@HA shell microneedle (TMH MN). 19.9 g GelMA‐β‐CD@QUE and 0.1 g lithium phenyl‐2,4,6‐trimethylbenzoylphosphinate (LAP) were dissolved in 100 mL ddH_2_O, and the mixture was cast into the microneedle mold under blue light irradiation (wavelength: 405 nm, duration: 60 s). Then, 3 cycles of vacuum injection were performed to eliminate internal and tip‐trapped bubbles, and the system was further dried at 37°C for 24 h before demolding to obtain QUE@GelMA core microneedle (QG MN).

The shell material (TA@MgO_2_@HA) was injected into the microneedle mold cavities and vacuum‐dried. The core material (QUE@GelMA) was then introduced into the cavities on top of the dried shell layer and subjected to further vacuum drying. Finally, the mold was removed to obtain the targeted core‐shell microneedles, designated as TMH/QG core‐shell microneedles.

### 5.4 Basic Properties of Core‐Shell Microneedles

The surface morphology of TA@MgO_2_@HA shell microneedle, QUE@GelMA core microneedle, and TMH/QG MN core‐shell microneedle samples were examined using scanning electron microscopy (SEM) carried out on an FEI Sirion 200 (operated at 30 kV). The bilayered architecture of the core‐shell microneedle arrays was visualized using CLSM (core: FITC fluorescent dye; shell: Cy5 fluorescent dye).

Dorsal skin regions of 8‐week‐old male Sprague‐Dawley (SD) rats (∼300 g) were depilated before microneedle application. At the end of the experiment, treated skin tissues were excised, immediately frozen in liquid nitrogen, and cryosectioned for hematoxylin and eosin (H&E) staining. Sections were imaged using an upright fluorescence microscope (Olympus BX63) and processed with CellSens Standard software.

Core‐shell microneedle (MN) arrays were vertically fixed in a 24‐well plate and submerged in PBS (pH 7.4) and PBS (pH 6.5). Bubble formation was monitored in real time using an inverted fluorescence microscope (Olympus IX83) with time‐lapse imaging throughout the experiment.

TA@MgO_2_@HA shell microneedle, QUE@GelMA core microneedle, and TMH/QG MN core‐shell microneedle samples were individually immersed in 5 mL of PBS (pH 7.4), PBS (pH 7.4 + H_2_O_2_), PBS (pH 6.5) and PBS (pH 6.5 + H_2_O_2_), respectively. After co‐incubation at 37°C for 24 h to reach swelling equilibrium, surface moisture was removed using filter paper, and the wet weight was recorded. Samples were then vacuum‐dried at 50°C to constant weight to obtain the dry weight. The swelling ratio was calculated as:

(1) $$\mathrm{Swelling}\;\mathrm{ratio}\;\left(\%\right)=\frac{W_{t}-W_{d}}{W_{d}}\times 100\%$$

Here, W_t_ and W_d_ represented the wet weight and the dry weight of sample, respectively.

TA@MgO_2_@HA shell microneedle, QUE@GelMA core microneedle and TMH/QG core‐shell microneedle samples were separately placed in PBS (pH 7.4), PBS (pH 7.4 + H_2_O_2_), PBS (pH 6.5) and PBS (pH 6.5 + H_2_O_2_), respectively. At scheduled periods (1, 4, 7, 14 and 21 days), samples were collected to measure the residual dry weight after removing the supernatant and drying overnight.

For photothermal characterization of the microneedle systems, TA@MgO_2_@HA shell microneedle, QUE@GelMA core microneedle, and TMH/QG MN core‐shell microneedle were separately placed in 2 mL microcentrifuge tubes with 0.5 mL of ddH_2_O and irradiated under the same conditions (1.5 W/cm^2^, 5 min), with continuous temperature monitoring. Additional comparative tests exposed the core‐shell microneedles to infrared fields at different power densities (1, 1.5 and 2 W/cm^2^). Cycling photothermal stability of TA@MgO_2_@HA shell microneedle, QUE@GelMA core microneedle, and TMH/QG MN core‐shell microneedle were similarly evaluated in 2 mL microcentrifuge tubes with 0.5 mL of ddH_2_O under high‐power irradiation (1.5 W/cm^2^). The system underwent 5 heating‐cooling cycles (5 min irradiation followed by natural cooling to ambient temperature), with temperature profiles recorded every 30 s. The TA@MgO_2_@HA shell microneedle, QUE@GelMA core microneedle, and TMH/QG MN core‐shell microneedle were also imaged sequentially under equivalent irradiation conditions in 2 mL microcentrifuge tubes containing 0.5 mL of ddH_2_O.

### 5.5 Mechanical Property of Core‐Shell Microneedles

The microneedle arrays were separated into individual needles (5–10 per test) for mechanical compression testing. Compression was performed using an IBTC‐300SL system at a constant rate of 0.02 mm/s until a displacement of 0.6 mm was reached. The compressive force per needle (N/needle) was calculated as:

(2) $$\mathrm{Comparassion}\;\mathrm{force}\left(N/\mathrm{needle}\right)=\frac{P}{n}$$

Here, P and n represented the compressive load and the number of needles, respectively.

Microneedle samples were cut into rectangular strips for tensile test. Tensile tests were conducted on the IBTC‐300SL system at a constant displacement rate of 0.02 mm/s until failure. Tensile strain and stress were calculated as follows:

(3) $$\varepsilon \left(\%\right)=\frac{L-L_{0}}{L_{0}}$$

(4) $$\sigma \left(\mathrm{Pa}\right)=\frac{P}{A}$$

Here, L is the length after deformation, L_0_ is the original gauge length, P is the tensile load, and A is the initial cross‐sectional area. The tensile modulus was determined from the slope of the linear elastic region of the stress‐strain curve.

### 5.6 ROS‐Resistant Capacity of Core‐Shell Microneedles

Total DPPH radical scavenging capacity was measured by observing the color alteration of the mixture solution containing DPPH and samples. All microneedle samples were mixed with pure DPPH solution for incubation at 37°C. After that, the mixture solution of each group was photographed to record the DPPH radical scavenging capacity, and the UV–vis absorption spectra were measured by a TU‐1810 ultraviolet‐visible spectrophotometer (Purkinje General Instrument Co. Ltd. Beijing, China) using QS‐grade quartz cuvettes at room temperature. These incubated solutions were collected to detect the absorption at 517 nm wavelength via the microplate reader. The DPPH radical clearance capacity was calculated as follows:

(5) $$\mathrm{DPPH}\;\mathrm{radical}\;\mathrm{clearance}\;\mathrm{capacity}\left(\%\right)=\frac{A_{C}-A_{s}}{A_{C}}*100\%$$

Here, A_c_ and A_s_ represented the absorption of the pure DPPH solution and the mixture solution containing DPPH and various samples, respectively.

The H_2_O_2_ Scavenging capacity and total SOD activity were determined using a commercial Hydrogen Peroxide Assay Kit (S0038, Beyotime) and a Total Superoxide Dismutase Assay Kit with WST‐8 (S0101M, Beyotime), following the provided protocol.

### 5.7 Cell Culture

AF tissues were carefully isolated from the IVDs of 8‐week‐old Sprague‐Dawley (SD) rats. The harvested tissues were minced and incubated in 0.1% collagenase I (Gibco, 17100017) solution for 40 min at 37°C, after which digestion was terminated with Dulbecco's Modified Eagle Medium/Nutrient Mixture F‐12 (DMEM/F‐12) (Gibco, C11330500BT). The resulting cell suspension was filtered through a 40 µm nylon cell strainer (Corning, 352340) to obtain a single‐cell suspension, which was washed with PBS to remove residual enzymes and cellular debris. Subsequently, the mixture was centrifuged at 1000 × g for 5 min, and the pellet was collected to seed primary AF cells into culture flasks containing DMEM/F‐12 medium. The cells were cultured in an incubator at 37°C with 5% CO_2_, and the medium was replaced regularly. Once the cells reached 80% confluency, third‐generation AF cells were utilized for subsequent experiments. AFCs were pretreated with TBHP for 1 h to establish an oxidative stress model, followed by co‐culturing with microneedles for therapeutic evaluation.

### 5.8 Cytocompatibility

AF cells were seeded into 12‐well plates at a density of 5 × 10^3^ cells/well and co‐cultured with the materials for 3 days. Live and dead cells were stained using the Calcein/PI Cell Viability and Cytotoxicity Assay Kit (Beyotime, C2015S), and observations were made using an inverted fluorescence microscope (Olympus IX83, China). The captured images were then subjected to quantitative analysis using ImageJ software.

### 5.9 Detection of MDA and GSH

The concentrations of malondialdehyde (MDA) and glutathione (GSH) were quantitatively determined using the Lipid Peroxidation MDA Assay Kit (Beyotime, S0131S) and GSH and GSSG Assay Kit (Beyotime, S0035), respectively. Briefly, the MDA concentration was directly quantified by converting the absorbance values of the samples using the corresponding standard curve. The GSH content was calculated using the following formula:

(6) GSH=Totalglutathione−GSSG×2

where the total glutathione content was the GSSG concentration calculated from the standard curve multiplied by 2, and the GSSG concentration referred to the concentration obtained after eliminating GSH.

### 5.10 Intracellular ROS Detection

AF cells were incubated with DCFH‐DA (10 µM) (Beyotime, S0033S) at 37°C for 30 min. Subsequently, the cells were washed thrice with serum‐free medium and observed under an inverted fluorescence microscope (Olympus IX83, China), with fluorescence intensity quantified using ImageJ software. Intracellular ROS levels were quantitatively analyzed using flow cytometry for verification.

### 5.11 Detection of Intracellular LPO and Fe^2+^

AF cells were exposed to serum‐free medium with Liperfluo (10 µM) (DOJINDO, L248) for Lipid Peroxidation (LPO) detection. After incubation at 37°C with 5% CO_2_ for 30 min, the solution was removed, the cells were washed three times with serum‐free medium, and visualized using an inverted confocal microscope (ZEISS LSM880, Germany). The fluorescence intensity was quantified using ImageJ software. Similarly, AF cells were exposed to 1 µM FerroOrange working solution (DOJINDO, F374) for Fe^2+^ detection. Then, the solution was removed after incubation at 37°C with 5% CO_2_ for 30 min. AF cells were washed three times with serum‐free medium and visualized using an inverted confocal microscope (ZEISS LSM880, Germany). The fluorescence intensity was quantified using ImageJ software.

### 5.12 Western Blotting

AF cells were collected and lysed on ice for 30 min with cell lysis buffer (Beyotime, P0013) supplemented with a protease inhibitor cocktail (Beyotime, P1005), followed by centrifugation at 10 000 × g for 15 min. Protein concentration was quantified using the BCA Protein Assay Kit (Genesand, PA101). The corresponding loading buffer was added to the protein solution, after which the solution was completely denatured by boiling in a water bath for 10 min. Equal amounts of protein were separated by polyacrylamide gel electrophoresis (TGX Stain‐Free FastCast Kit, BIO‐RAD, 1610183) at 120 V and transferred onto polyvinylidene fluoride (PVDF) membranes (Beyotime, FFP39). Following blocking with protein‐free rapid blocking solution (Servicebio, G2052‐500ML), the membranes were incubated overnight at 4°C with primary antibodies (1:1000 dilution) against ACSL4 (proteintech, 22401‐1‐AP), GPX4 (Abclonal, A1933), MMP3 (proteintech, 17873‐1‐AP), MMP13 (Abclonal, A11148), COL1 (proteintech, 14695‐1‐AP), DNMT1 (proteintech, 24206‐1‐AP), DNMT3A (proteintech, 20954‐1‐AP), DNMT3B (proteintech, 26971‐1‐AP), LaminB1 (proteintech, 12987‐1‐AP), ATF3 (Abclonal, A13469) and β‐actin (BOSTER, BA2305). Thereafter, the membranes were incubated with the corresponding HRP‐conjugated secondary antibodies, and chemiluminescent detection was performed using the Enhanced ECL Chemiluminescence Kit (BeyoECL Plus, Beyotime, P0018S). Images were acquired using a standard gel imaging system (Bio‐Rad, USA) and subjected to quantitative analysis using ImageJ software. The corresponding uncropped blot images are provided in Table S1.

### 5.13 Quantitative Real‐Time PCR

Total RNA was extracted from the samples using the Fastagen Total RNA Isolation Kit (Fastagen, 220011), and the quantity and purity of the extracted RNA were assessed using a NanoDrop ONEc Microvolume UV–vis Spectrophotometer. Subsequently, the mRNA of the target genes was reverse‐transcribed into cDNA by adding the specified reagents according to the instructions of the RevertAid Master Mix (Thermo Fisher, M16325). Quantitative analysis of cDNA was performed by RT‐qPCR using SYBR Green qPCR SuperMix (Thermo Fisher, 11744500). The expression levels of the target genes were quantified using the ΔΔCt method, with the relative expression of each gene calculated using the 2^‐∆∆Ct^ formula. The primer sequences used are listed in Tables S2 and S3.

### 5.14 Lentiviral Transfection

Vector plasmid construction was performed by Tsingke Biotechnology Co., Ltd. A plasmid mixture (7.5 µg vector plasmid + 5.6 µg psPAX2 + 1.9 µg pMD2.G) was added to 500 µL of serum‐free DMEM and gently mixed. Separately, 45 µL of PEI (1 mg/mL, Yeasen, 40816ES01) was added to another tube containing 500 µL of serum‐free DMEM, mixed thoroughly, and allowed to stand at room temperature for 1 min. Following this, the PEI solution was added dropwise to the DNA solution, vortexed for 10 s with sealing, and incubated at room temperature for 15–20 min to form DNA‐PEI complexes. Human embryonic kidney cells (HEK293T) seeded in 100 mm cell culture dishes at a density of 6 × 10^6^ cells/dish were removed from the incubator, the old medium was aspirated, 8 mL fresh complete medium was added, and 1 mL DNA‐PEI complexes were added dropwise and mixed gently by swirling. After 6 h of incubation, the medium was replaced with 10 mL of fresh complete medium. At 48 h post‐transfection, the culture supernatant containing viral particles was aspirated with a sterile pipette, filtered through a 0.45 µm PVDF membrane to remove cell debris, and 10 mL of fresh complete medium was added to the cells for continued culture. Following 72 h of transfection, the above filtration step was repeated, and the viral supernatants collected twice were combined, aliquoted, and stored at −80°C for subsequent use. For transduction, AF cells were seeded in 6‐well plates at a density of 1 × 10^5^ cells/well and cultured overnight. For transduction, the frozen viral solution was rapidly thawed on ice, and the calculated volumes of viral solution, polybrene (10 mg/mL stock solution, Yeasen, 40804ES76), and complete medium were added to each well to ensure a final polybrene concentration of 8 µg/mL and multiplicity of infection (MOI) of 10. After 12 h, the medium was replaced with fresh complete medium. Following a 72‐h post‐transduction gene regulation period, successful gene regulation was confirmed at the protein level using western blotting.

### 5.15 mRNA Stability Assay

mRNA stability was evaluated following the administration of actinomycin D (Selleck, S8964). AF cells were treated with 5 µg/mL Actinomycin D, and total mRNA was extracted at 0, 0.5, 1, 2 and 4 h post‐treatment. The mRNA levels of ATF3 were detected using RT‐qPCR.

### 5.16 Methylation‐Specific PCR (MSP)

The *Atf3* promoter region was retrieved from the UCSC Genome Browser (http://genome.ucsc.edu/), and CpG islands were predicted using the online MetPrimer tool (https://www.urogene.org/methprimer), based on which primers for MSP were designed. The primer sequences used are listed in Tables S2 and S3. Genomic DNA was extracted from the samples using a DNA Extraction Kit (TIANGEN, DP304‐02), and the concentration of the extracted DNA was determined. Bisulfite conversion of the extracted DNA samples was performed using the DNA Bisulfite Conversion Kit (Beyotime, D0068S), and PCR was performed using the Methylation‐Specific PCR Kit (TIANGEN, EM101‐01). The PCR cycling program utilized in this study (20 µL system) is outlined below: Following a brief centrifugation step, the reaction mixture was transferred into a PCR thermocycler and subjected to pre‐denaturation at 95°C for 10 min, followed by 35 cycles of denaturation at 95°C for 15 s, annealing at 58°C for 15 s, and extension at 72°C for 30 s, and a final extension at 72°C for 10 min, after which the products were stored at 4°C. PCR products were analyzed by agarose gel electrophoresis: 1.5% agarose gel (Solarbio, A8201) was prepared, 8 µL of PCR product mixed with loading buffer was loaded into each gel well, and 8 µL GoldBand DL600 DNA Marker (Yeasen, 10502ES60) was loaded. Electrophoresis was performed at 120 V for 20 min, after which images were obtained using a standard gel imaging system (Bio‐Rad, USA), and analysis was conducted using Image Lab software. The methylation levels were quantified by densitometric analysis of agarose gel bands using ImageJ, with the methylation percentage calculated as the ratio of the methylated band intensity to the sum of the methylated and unmethylated band intensities. The corresponding uncropped gel images are provided in Table S1.

### 5.17 Animal Model Establishment

All experimental procedures involving animals were performed in strict accordance with institutional and national ethical guidelines and were reviewed and approved by the Experimental Animal Ethics Committee of the Third Military Medical University (Chongqing, China). 9‐week‐old adult male SD rats (SPF grade) were procured from the Experimental Animal Center of the Third Military Medical University and randomly divided into four groups (Con), IDD, core‐shell TMH/QG MN and core‐shell TMH/QG microneedles combined with NIR irradiation. Following anesthesia induction with 1% sodium pentobarbital (4 mL/kg) via intraperitoneal injection, a 21G puncture needle was inserted vertically into the caudal vertebral endplate and rotated for 30 s to establish an AF injury model. Core‐shell TMH/QG MN were inserted into the surface of the AF, and the wound was sutured. Rats in the experimental group were subjected to NIR light irradiation therapy on a bi‐daily basis postoperatively (5 min per session). Caudal IVD tissue samples were collected at 4 and 8 weeks postoperatively for comprehensive histological and imaging analyses.

### 5.18 Degradation Experiment In Vivo

8‐week‐old adult male SD rats were randomly divided into five groups. After anesthesia with 1% sodium pentobarbital, a 21G puncture needle was inserted into the caudal vertebrae, rotated for 30 s, and withdrawn to establish an AF injury model. TMH MN, QG MN and core‐shell TMH/QG MN were inserted into the surface of the AF, after which the wound was sutured. Rats in the experimental group received NIR light irradiation therapy every other day postoperatively (5 min per session). The in vivo degradation behavior of the MNs was monitored using a small animal in vivo imaging system at 0, 7, 14, and 21 days postoperatively.

### 5.19 Photothermal Imaging In Vivo

After implanting the different MN samples into the rat IVD puncture model with NIR laser irradiation treatment, the in vivo temperature field distribution was monitored using an infrared thermal imager, and analysis was performed using FLIR Tools software.

### 5.20 Radiological Analysis

At 4 and 8 weeks post‐surgery, the rats underwent X‐ray and MRI assessments. The disc height index (DHI) was calculated as a percentage (DHI%) and compared with normal disc measurements on X‐ray. The MRI evaluation of disc degeneration was conducted using Pfirrmann grading on T2‐weighted images.

### 5.21 Histological Evaluation

The collected IVD samples were fixed with 4% paraformaldehyde and decalcified with 14% EDTA for 1 month. Decalcified IVDs were embedded in paraffin and sectioned to a thickness of 2.5 µm. Hematoxylin‐Eosin (HE) staining and Safranin O‐Fast Green staining were performed to observe structural changes of the IVD. Immunohistochemical staining was utilized to detect the expression of COL1 (proteintech, 14695‐1‐AP, 1:500), MMP3 (proteintech, 17873‐1‐AP, 1:100), ATF3 (Abclonal, A13469, 1:100), ACSL4 (proteintech, 22401‐1‐AP, 1:100), GPX4 (Abclonal, A1933, 1:100), and DNMT3A (proteintech, 20954‐1‐AP, 1:500) in the AF.

### 5.22 Immunohistochemical Staining

Frozen sections were air‐dried at room temperature and baked in an oven at 37°C for 30 min, followed by washing three times with PBS. A 3% hydrogen peroxide solution was added dropwise, and the sections were incubated at room temperature in the dark for 25 min. Protein‐free rapid blocking solution (Servicebio, G2052‐500ML) was added to cover the tissue uniformly, and blocking was performed at room temperature for 30 min. Following the removal of the blocking solution, primary antibodies diluted with PBS (ACSL4 (Proteintech, 22401‐1‐AP), GPX4 (Abclonal, A1933) and ATF3 (Abclonal, A13469), all at 1:100 dilution) were added dropwise to the sections, which were then placed in a humidified chamber and incubated overnight at 4°C. Secondary antibodies corresponding to the species of the primary antibodies were added to the tissue at room temperature for 50 min. Freshly prepared DAB chromogenic solution (ZSGB‐BIO, ZLI‐9018) was added, and the chromogenic reaction was performed at room temperature for 5 min. Hematoxylin (Servicebio, G1004‐100ML) counterstaining was performed for 5 min, followed by differentiation with hematoxylin differentiation solution (Servicebio, G1039‐500ML) for 30 s and bluing with hematoxylin bluing solution (Servicebio, G1040‐500ML) for 2 min. After dehydration and clearing, the sections were mounted using Van‐Mount environmentally friendly mounting medium (Huntz, H‐H0401). Subsequent Image acquisition and analysis were performed using a VS200 whole‐slide scanning imaging system (VS200, Olympus, China).

### 5.23 Mechanical Property Testing of IVD Samples

IVD were harvested from rat tails 8 weeks postoperatively, and their original cross‐sectional area and height were measured. The compressive properties of all samples were measured using a micro multiscale in situ mechanical testing system (IBTC‐300SL) at a speed of 0.2 mm/s. The corresponding compressive modulus was calculated as follows:

(7) $$\varepsilon \left(\%\right)=\frac{-\left(L-L_{0}\right)}{L_{0}}*100\%$$

(8) $$\sigma \left(\mathrm{Pa}\right)=\frac{P}{A}$$

Here ε, σ were the compression strain and stress, respectively. L, L_0_, P, and A were the length of IVD samples after compression, the original length of the IVD samples, compression load and the original cross‐sectional area of the IVD samples, respectively. The compressive modulus was obtained by calculating the gradient of the linear elastic stage of the compressive stress‐strain curve.

To investigate the fatigue resistance of the repaired IVDs, IVD samples harvested from rat tails at 8 weeks postoperatively were recorded for their original cross‐sectional area and height. The fatigue resistance of all samples was tested by compressing them to 10% strain at a rate of 0.2 mm/s for 15 cycles using the IBTC‐300SL micro multi‐scale in situ mechanical testing system.

### 5.24 Statistical Analysis

Data are presented as mean ± standard deviation (SD), and each experiment was repeated at least three times. Statistical analyses were conducted using GraphPad Prism 10.11.2 and Origin 2024. Comparisons between two groups were performed using Student's *t*‐test in GraphPad Prism 10.11.2, and one‐way analysis of variance (ANOVA) followed by Tukey's test was used for multiple group comparisons in Origin 2024. For non‐normally distributed data, non‐parametric tests were performed using GraphPad Prism 9 software. Statistical significance was defined as *p* < 0.05.

## Author Contributions

L.T., Y.W., and M.L. designed the study. L.T., and Y.Z. performed the experiments and acquired the data. L.T., Y.Z., Y.L., Z.C., Y.W., and S.W. analyzed the data. Y.W., and M.L. collected clinical samples. L.T., Y.W., and Y.Z. wrote this manuscript. C.L., Y.W., and M.L. supervised the study.

## Funding

This study was supported by the National Natural Science Foundation of China (No. 82502900 for L. Tan, No. 82472445 for M. Liu), Chongqing Municipal Natural Science Foundation (No. CSTB2025NSCQ‐GPX0589 for L. Tan, No. CSTB2025NSCQ‐GPX0056 for Y. Wang), Chongqing Special Funding for Postdoctoral Research Projects (2025CQBSHTB3010), Young Doctoral Talent Cultivation Program of Xinqiao Hospital (2025YQB004), and Discipline Talent Construction Project of Xinqiao Hospital (2025XKRC013).

## Conflicts of Interest

The authors declare no conflicts of interest.

## Supporting information




**Supporting File 1**: advs77829‐sup‐0001‐SuppMat.docx.

**Supporting File 2**: advs77829‐sup‐0002‐TableS1.xlsx.

## Data Availability

All data needed to evaluate the conclusions in the paper are present in the paper and/or the Supplementary Materials. Additional data related to this paper may be requested from the authors.
