# Supplementary material for: Acid‐Responsive Nanobot‐Integrated Core‐Shell Microneedles Reprogram the Degenerative Annulus Fibrosus Microenvironment Through Epigenetic Suppression of Ferroptosis
Source: Adv Sci (Weinh). 2026 Sep 27:e77829. Online ahead of print. doi: 10.1002/advs.77829 (PMC13616280; doi:10.1002/advs.77829)
Supplement: Supplementary file 1 [file ADVS-9999-e77829-s002.docx]

Supporting Information

Acid-Responsive Nanobot-Integrated Core-Shell Microneedles Reprogram the Degenerative Annulus Fibrosus Microenvironment through Epigenetic Suppression of Ferroptosis

*Lu Tan, Yan Zheng, Yue Lan, Zhuo Cheng, Siya Wang, Changqing Li, Yanqiu Wang*, and Minghan Liu**

L. Tan, Z. Cheng, S. Wang, C. Li, Y. Wang, M. Liu

Department of Orthopedics, Xinqiao Hospital, Army Medical University, Chongqing 400037, China.

E-mail: wangyanqiu@tmmu.edu.cn; liuminghan@tmmu.edu.cn

L. Tan, Z. Cheng, S. Wang, C. Li, Y. Wang, M. Liu

Chongqing Municipal Health Commission Key Laboratory of Precise Orthopedics.

E-mail: wangyanqiu@tmmu.edu.cn; liuminghan@tmmu.edu.cn

Y. Zheng

Chongqing Yuyue Scientific Research Center for Pathology, Jinfeng Laboratory, Chongqing 401329, China.

Y. Lan

School of Pharmacy and Bioengineering, Chongqing University of Technology, Chongqing 400054, China.

Y. Wang

Department of Orthopedics, Army 953 Hospital, Shigatse Branch of Xinqiao Hospital, Army Medical University, Shigatse, Tibet Autonomous Region 857000, China.

**Figures and Captions**

**Figure S1.** (A) UV-vis spectra of TA solutions at different concentrations. (B) UV-vis spectra of TA, MgO_2_ and TA@MgO_2_ composites. (C) Standard curve of TA. (D) Drug loading capacity of TA@MgO_2_ composites prepared with different initial mass ratios of MgO_2_ to TA. (E) Quantitative temperature statistics of TA, MgO_2_ and TA@MgO_2_ under NIR irradiation. (F) Photothermal heating curves of TA@MgO_2_ (1 mg/mL) under NIR irradiation at different power densities (1.0, 1.5, and 2.0 W/cm^2^). (G) Quantitative evaluation of the multi-enzyme mimetic activities of TA@MgO_2_: DPPH radical scavenging capacity at different concentrations. Data are presented as mean ± SD (n ≥ 3), and *p*-values were calculated using one-way ANOVA with Tukey's multiple comparisons test [(D), (E) and (G)].

**Figure S2.** (A) Schematic illustrations of the synthetic routes for HA-ADH-HCA. (B) Schematic illustrations of the synthetic routes for GelMA-β-CD@QUE.

**Figure S3.** (A) FTIR spectra of HA, ADH, HA-ADH and HA-ADH-HCA. (B) FTIR spectra of Gelatin and GelMA. (C) FTIR spectra of β-CD and β-CD-COOH, respectively. (D) FTIR spectra of GelMA, CM-β-CD and GelMA-β-CD composites.

**Figure S4.** (A-D) ^1^H NMR spectra of HA, ADH, HA-ADH and HA-ADH-HCA. (E-H) ^1^H NMR spectra of Gelatin, GelMA, β-CD and GelMA-β-CD.

**Figure S5.** (A) UV-vis absorption spectra of QUE solutions at different concentrations, showing a characteristic peak at 375 nm with no obvious shift in peak position. (B) UV-vis spectra of QUE, GelMA-β-CD and GelMA-β-CD@QUE composites. (C) Standard curve of QUE used for quantitative analysis. (D) Drug loading capacity of GelMA-β-CD@QUE composites prepared with different initial mass ratios of GelMA-β-CD to QUE. (E) Photothermal heating curves of TMH/QG MN under NIR irradiation at different power densities (1.0, 1.5 and 2.0 W/cm^2^). (F) Quantitative temperature statistics of QG, TMH and TMH/QG MNs under NIR irradiation. Data are presented as mean ± SD (n ≥ 3), and *p*-values were calculated using one-way ANOVA with Tukey's multiple comparisons test [(D) and (F)].

**Figure S6.** (A) Quantitative analysis of height changes in TMH/QG MN in four simulated microenvironments at 0, 7 and 14 days. (B) Degradation rate of TMH/QG MN in four simulated microenvironments. (C) Degradation rate of TMH/QG MN under four simulated cell-culture microenvironments. (D-E) Cumulative release profiles of TA and QUE from TMH/QG MN in four simulated microenvironments. (F) Quantitative assessment of antioxidant activities: DPPH radical scavenging rate, SOD-like activity and H_2_O_2_ scavenging capacity of different MN groups. Data are presented as mean ± SD (n ≥ 3), and *p*-values were calculated using one-way ANOVA with Tukey's multiple comparisons test [(A) and (D) to (F)].

**Figure S7.** (A-C) Quantitative analysis of the mechanical properties of QG, TMH and TMH/QG MNs under tensile testing: tensile modulus, fracture strength and elongation at break. (D) Digital photographs of AF penetrated by the core-shell TMH/QG MN array. Data are presented as mean ± SD (n ≥ 3), and *p*-values were calculated using one-way ANOVA with Tukey's multiple comparisons test [(A) to (C)].

**Figure S8.** (A) Live/dead staining images of normal AFCs after 1-day and 3-day co-culture with QG, TMH and TMH/QG MNs with or without NIR irradiation. Scale bar: 100 μm. (B) Bright-field micrographs of normal AFCs cultured under the above-described conditions. Scale bar: 100 μm. (C) Quantitative analysis of live-cell fractions for normal AFCs upon 1- and 3-day co-culture with QG, TMH, and TMH/QG MNs with or without NIR irradiation. (D) Cell viability of TBHP-pretreated AFCs after co-culturing with or without the indicated MNs under NIR irradiation. (E) Relative mRNA expression levels of *Acsl4*, *Gpx4*, *Col1α1* and *Mmp3* in TBHP-pretreated AFCs with or without MN treatment under NIR irradiation. Data are presented as mean ± SD (n ≥ 3), and *p*-values were calculated using one-way ANOVA with Tukey's multiple comparisons test [(C) to (E)].

**Figure S9.** (A) Cluster analysis of DEGs between TBHP+NIR and TBHP+NIR+TMH/QG groups. (B-C) GSEA plots demonstrating significant enrichment of DEGs in the MAPK signaling pathway, the ERK1/ERK2 cascade, extracellular matrix degradation and immune response. (D) Relative mRNA expression levels of *Atf3* and *Trib2.* (E) Corresponding quantitative statistics of ATF3 and TRIB2 protein expression levels in TBHP+NIR and TBHP+NIR+TMH/QG groups. (F-G) Western blot analysis and corresponding quantification of ATF3 expression in AFCs with ATF3 knockdown (shAtf3_1, shAtf3_2 and shAtf3_3) or overexpression (LV-Atf3). The corresponding uncropped blot images are provided in Table S1. (H) Western blot analysis and corresponding quantification of ACSL4 and GPX4 in the shAtf3 group. The corresponding uncropped blot images are provided in Table S1. (I) Western blot analysis and corresponding quantification of ACSL4 and GPX4 in LV-Atf3 group. The corresponding uncropped blot images are provided in Table S1. (J) Quantitative analysis of C11-BODIPY staining to assess lipid peroxidation in shAtf3 and LV-Atf3 groups. (K) Comparison of nuclear ATF3 levels between LV-Atf3 and LV-NC control groups after TBHP+TMH/QG+NIR treatment. (L) Corresponding quantitative statistics of ECM (COL1, MMP3 and MMP13) and ferroptosis (ACSL4 and GPX4) markers in TBHP+TMH/QG+NIR+LV-Atf3 and TBHP+TMH/QG+NIR+LV-NC groups. (M) Measurement of MDA content in TBHP+TMH/QG+NIR+LV-Atf3 and TBHP+TMH/QG+NIR+LV-NC groups. (N) Quantification of intracellular ROS levels in TBHP+TMH/QG+NIR+LV-Atf3 and TBHP+TMH/QG+NIR+LV-NC groups. (O-P) Corresponding fluorescence intensity of BODIPY and FerroOrange in TBHP+TMH/QG+NIR+LV-Atf3 and TBHP+TMH/QG+NIR+LV-NC groups. Data are presented as mean ± SD (n ≥ 3), and *p*-values were calculated using two-tailed Student's *t*-test [(D) to (E) and (K) to (P)] and one-way ANOVA with Tukey's multiple comparisons test [(F) to (J)].

**Figure S10.** (A) Relative *Atf3* mRNA expression levels in the TBHP+NIR and TBHP+NIR+TMH/QG groups after treatment with Actinomycin D (5 μg/mL) for the indicated times. (B) GSEA plots show significant enrichment of DEGs in the DNA repair pathway. (C) Schematic representation of the rat *Atf3* promoter region, indicating five high-density CpG islands (CGIs). (D) Quantification of DNA methyltransferases (DNMT1, DNMT3A and DNMT3B) in the TBHP+NIR and TBHP+NIR+TMH/QG groups. (E-F) Immunofluorescence staining and fluorescence intensity quantification of DNMT3A expression in the TBHP+NIR and TBHP+NIR+TMH/QG groups. Scale bar: 20 μm. (G) Immunofluorescence staining and fluorescence intensity quantification of SLC7A11 expression in the TBHP+NIR+TMH/QG group with or without DY-46-2. Scale bar: 20 μm. (H) Quantification of intracellular ROS levels. (I) Quantification of SLC7A11 and ATF3 protein levels in the TBHP+NIR+TMH/QG group with or without DY-46-2. (J) Quantification of FerroOrange in the TBHP+NIR+TMH/QG group with or without DY-46-2. (K) Quantification of key ferroptosis (ACSL4 and GPX4) and extracellular matrix (COL1, MMP3 and MMP13) markers in the TBHP+NIR+TMH/QG group with or without DY-46-2. (L) Schematic representation of the human *ATF3* promoter region, indicating five high-density CpG islands. (M) Corresponding qualification of the ATF3 promoter methylation level in human mild and severe degeneration groups. (N-P) Quantification of ATF3, SLC7A11 and DNMT3A expression in human disc tissues from mild and severe degeneration groups. (Q-R) Relative protein levels and mRNA expression of DNMT3A, SLC7A11, ATF3, and *DNMT3A*, *SLC7A11*. Data are presented as mean ± SD (n ≥ 3), and *p*-values were calculated using two-tailed Student's *t*-test [(D), (E), (G) to (K), and (M) to (R)] and one-way ANOVA with Tukey's multiple comparisons test [(A)].

**Figure S11.** (A) Representative H&E-stained images and corresponding semi-quantitative scoring of major organs (heart, liver, spleen, lungs, and kidneys) of SD rats in the Con, IDD, TMH/QG, and TMH/QG+NIR groups. Scale bar: 50 μm. (B) Hematological parameters of rats in the Con, IDD, TMH/QG, and TMH/QG+NIR groups (WBC: white blood cells, HGB: hemoglobin, RBC: red blood cells, MCV: mean corpuscular volume, MCH: mean corpuscular hemoglobin, PLT: platelets). (C) Quantitative temperature profiles of rat AF treated with QG, TMH and TMH/QG MNs under NIR irradiation (1.5 W/cm^2^). (D) In vivo degradation profiles of microneedles at different time points. (E) Representative H&E histological scores. (F) Relative protein levels of ATF3, DNMT3A and ferroptosis markers (ACSL4 and GPX4) in disc tissues. (G) Relative mRNA expression levels of *Atf3*, *Dnmt3a*, *Acsl4* and *Gpx4* in disc tissues across the different treatment groups. (H) Positive area of ATF3 in AF tissues. (I-M) Positive area of DNMT3A, ACSL4, GPX4, COL1 and MMP3 in AF tissues. Data are presented as mean ± SD (n ≥ 3), and p-values were calculated using one-way ANOVA with Tukey's multiple comparisons test [(B) to (C) and (F) to (M)] and Kruskal-Wallis test with Dunn's multiple comparisons test [(A) and (E)].

**Figure S12.** (A) Representative stress-strain curves of IVDs from the Con, IDD and TMH/QG+NIR groups, measured immediately after treatment. (B) Energy dissipation efficiency of IVDs from Con, IDD and TMH/QG+NIR groups. (C) Fatigue resistance of IVDs evaluated by 15 consecutive cyclic compression tests from Con, IDD and TMH/QG+NIR groups. Data are presented as mean ± SD (n ≥ 3), and *p*-values were calculated using one-way ANOVA with Tukey's multiple comparisons test [(B)].

**Table S2.** Sequences of primers used for PCR analysis of Rat.

| **Name** | **Forward (5' - 3')** |
| --- | --- |
| *Atf3* | Forward: GAAGATGAGAGGAAAAGGAGGCG |
|  | Reverse: GCTCAGCATTCACACTCTCCAG |
| *Atf3-CGI1 M* | Forward: TTGGTTATTAATTTTAGGTTGACGT |
|  | Reverse: GAATACGCACATCTAACGATACG |
| *Atf3-CGI1 U* | Forward: TAATTTTTGGTTATTAATTTTAGGTTGATG |
|  | Reverse: CAAATACACACATCTAACAATACACC |
| *Atf3-CGI2 M* | Forward: TTTTCGTTTATAGTTGTGGGTTAAC |
|  | Reverse: ACTAACACCTTAAATTCCTAACGAA |
| *Atf3-CGI2 U* | Forward: TTGTTTATAGTTGTGGGTTAATGT |
|  | Reverse: ACTAACACCTTAAATTCCTAACAAA |
| *Atf3-CGI3 M* | Forward: ACGTATCGTTTAAAGTCGGAATATC |
|  | Reverse: CGAAATCACTAAATCAAAATCGAA |
| *Atf3-CGI3 U* | Forward: TGTATTGTTTAAAGTTGGAATATTGG |
|  | Reverse: TCAAAATCACTAAATCAAAATCAAA |
| *Atf3-CGI4 M* | Forward: TATAGTATTTGTTATTCGGACGGTTAC |
|  | Reverse: GAATACAAAAAACTTAATTTTACCCGA |
| *Atf3-CGI4 U* | Forward: TATAGTATTTGTTATTTGGATGGTTATGG |
|  | Reverse: AAATACAAAAAACTTAATTTTACCCAAA |
| *Atf3-CGI5 M* | Forward: TTTTTTGATTTTGTTAGGATTTGAC |
|  | Reverse: CGAATTTACCTTAAAACAAATCGAC |
| *Atf3-CGI5 U* | Forward: GATTTTGTTAGGATTTGATGG |
|  | Reverse: CAAATTTACCTTAAAACAAATCAAC |
| *DNMT1* | Forward: ACCGGCAGGTGGGGAA |
|  | Reverse: CTGCAGCTGAGGCACTCTC |
| *DNMT3A* | Forward: CAGCTATTTACAGAGCTTCGGG |
|  | Reverse: CTTGGCTTTCTTCTCAGCCCAT |
| *DNMT3B* | Forward: AGGAAGGACTGGAACATGCG |
|  | Reverse: ATCGCTGGGTACAACTTGGG |
| *LMNB1* | Forward: CAGATTGCCCAGTTGGAAGC |
|  | Reverse: GCAAAGTCTCATCCGCCAAC |

**Table S3.** Sequences of primers used for PCR analysis of Human.

| **Name** | **Primer (5'>3')** |
| --- | --- |
| *ATF3* | Forward: CCTCTGCGCTGGAATCAGTC |
|  | Reverse: TTCTTTCTCGTCGCCTCTTTTT |
| *ATF3-CGI1 M* | Forward: AGTAAAGCGAAAATATTCGT |
|  | Reverse: GAAAACCCTAAAATTCCACGC |
| *ATF3-CGI1 U* | Forward: GAAAAGTAAAGTGAAAATATTTGT |
|  | Reverse: CAAAAACCCTAAAATTCCACAC |
| *ATF3-CGI2 M* | Forward: TAGTTTTGTTGAGGTTTTAGTTCGA |
|  | Reverse: GTATTCTTCTACTCCCAAAACCGA |
| *ATF3-CGI2 U* | Forward: TAGTTTTGTTGAGGTTTTAGTTTGA |
|  | Reverse: ACATATTCTTCTACTCCCAAAACCA |
| *ATF3-CGI3 M* | Forward: ATTATTCGTTTTAGTTTCGGAGTTC |
|  | Reverse: AATATCCAAAATCACCCTTCGAT |
| *ATF3-CGI3 U* | Forward: TATTTGTTTTAGTTTTGGAGTTTGG |
|  | Reverse: AAATATCCAAAATCACCCTTCAAT |
| *ATF3-CGI4 M* | Forward: CGTTTTTTTCGTTTTTTAATTTAGC |
|  | Reverse: CCAAATATACGTACTCGCTACTACGTA |
| *ATF3-CGI4 U* | Forward: TTTGTTTTTTTTGTTTTTTAATTTAGTG |
|  | Reverse: CCAAATATACATACTCACTACTACATA |
| *ATF3-CGI5 M* | Forward: TTTTTTTTATTCGTTTTCGGTTTC |
|  | Reverse: CGTAATACTATTAATAACTTCCCGAA |
| *ATF3-CGI5 U* | Forward: TTTTTTTATTTGTTTTTGGTTTTGT |
|  | Reverse: ACATAATACTATTAATAACTTCCCAAA |
